# Investigation of Leoligin Derivatives as NF-κΒ Inhibitory Agents

**DOI:** 10.3390/biomedicines10010062

**Published:** 2021-12-28

**Authors:** Thomas Linder, Eleni Papaplioura, Diyana Ogurlu, Sophie Geyrhofer, Scarlet Hummelbrunner, Daniel Schachner, Atanas G. Atanasov, Marko D. Mihovilovic, Verena M. Dirsch, Michael Schnürch

**Affiliations:** 1 Institute for Applied Synthetic Chemistry, TU Wien, Getreidemarkt 9/163, 1060 Vienna, Austria; thomas.linder@thermofisher.com (T.L.); eleni.papaplioura@tuwien.ac.at (E.P.); sophie.geyrhofer@gmail.com (S.G.); marko.mihovilovic@tuwien.ac.at (M.D.M.); 2 Department of Pharmaceutical Sciences, University of Vienna, Althanstraße 14, 1090 Vienna, Austria; diyanaogurlu@hotmail.com (D.O.); scarlet.hummelbrunner@univie.ac.at (S.H.); daniel.schachner@univie.ac.at (D.S.); atanas.atanasov@univie.ac.at (A.G.A.); 3 Ludwig Boltzmann Institute for Digital Health and Patient Safety, Medical University of Vienna, Spitalgasse 23, 1090 Vienna, Austria; 4 Institute of Genetics and Animal Biotechnology of the Polish Academy of Sciences, Jastrzebiec, 05-552 Magdalenka, Poland

**Keywords:** natural product synthesis, lignans, inflammation, NF-κB inhibition

## Abstract

The transcription factor NF-κB is an essential mediator of inflammation; thus, the identification of compounds that interfere with the NF-κB signaling pathway is an important topic. The natural products leoligin and 5-methoxyleoligin have served as a starting point for the development of NF-κB inhibitors. Using our modular total synthesis method of leoligin, modifications at two positions were undertaken and the effects of these modifications on the biological activity were investigated. The first modification concerned the ester functionality, where it was found that variations in this position have a significant influence, with bulky esters lacking Michael-acceptor properties being favored. Additionally, the substituents on the aryl group in position 2 of the tetrahydrofuran scaffold can vary to some extent, where it was found that a 3,4-dimethoxy and a 4-fluoro substitution pattern show comparable inhibitory efficiency.

## 1. Introduction

**Figure 1:**
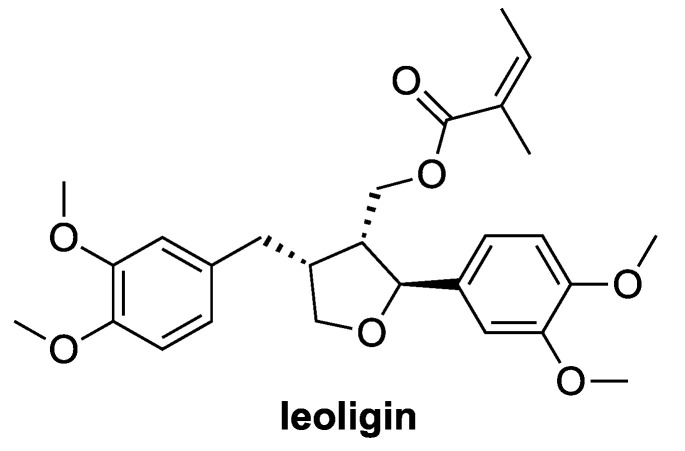
Structure of leoligin.

Leoligin [1] (Figure 1), a naturally occurring furan-type lignan, found in the roots of Edelweiss (*Leontopodium nivale* ssp. *alpinum*), was shown to display a pharmacological profile that suggests an overall anti-inflammatory activity. Reisinger et al. demonstrated that leoligin is able to reduce intimal hyperplasia and to decrease TNF-α (tumor necrosis factor α)-induced vascular cell adhesion molecule (VCAM)-1 expression in primary human endothelial cells [2], which is highly regulated by the nuclear factor kappa-light-chain-enhancer of activated B-cells (NF-κB) [3]. Leoligin was also identified as an inducer of macrophage cholesterol efflux, an activity that renders it a promising candidate in atherosclerosis-related experimental models [4]. 

In the context of a multi-disciplinary project on the synthesis of furan-type lignans with anti-inflammatory activity, several leoligin analogs were synthesized in our group and subjected to cell-based assays, which led to analogs that selectively inhibited VSMC (vascular smooth muscle cell) versus EC (endothelial cell) proliferation, which is advantageous in the treatment of vascular neointima formation [5]. Based on this finding and taking into account that drug-eluting stents releasing immunosuppressant drugs are able to reduce local VSMC proliferation, leoligin and derivatives thereof were exploited in drug-releasing experiments using an inexpensive stent model, aiming to determine their relative releasing properties [6]. Additionally, particular emphasis has been put on 5-methoxyleoligin, an interesting lignan that induces CYP26B1-dependent angiogenesis in vitro, and arteriogenesis in vivo, which are important benefits for post-myocardial infarction therapy [7]. 

The nuclear factor-κB (NF-κB) family of transcription factors consists of several protein members: NF-κB1 p50, NF-κB2 p52, p65 (also called RelA), RELB and c-REL [8]. NF-κB exists as an inactive complex bound to members of the inhibitor of κB (IκB) protein family. Proinflammatory stimuli, such as cytokines, activate the NF-κB signaling cascade, which leads to proteasomal degradation of the IκB protein, release of the transcription factor and its translocation into the nucleus, where it binds to respective response elements in the DNA as a dimeric complex [8]. Target genes regulated by NF-κB include a wide variety of proinflammatory genes. Thus, NF-κB plays a fundamental role in mediating all classical attributes of inflammation [9]. Hence, suppression of NF-κB is a topic of significant interest in medicinal chemistry. Over the years, a plethora of inhibitors has been identified with different mechanisms interfering with the NF-κB signaling pathway. They range from proteasome inhibitors such as bortezomib to IKK inhibitors such as parthenolide. Additionally, established drugs such as acetylsalicylic acid are reported to act as NF-κB inhibitors [10,11,12].

Leoligin itself is a weak inhibitor of the NF-κB pathway. It is therefore interesting to investigate how modifications to the parent leoligin motif would impact this activity. Within a previous study, we studied leoligin and a small set of leoligin analogs to identify structural features [4,5,6,13], that can improve either NF-κB inhibition or inhibition of the proliferation of VSMCs [5]. The present contribution is dedicated to reveal the structure–activity relationships of leoligin-like lignans affecting NF-κB. Herein, we report our investigations towards modifications of the leoligin motif that might impact such an activity.

## 2. Materials and Methods

### 2.1. General Information

Unless noted otherwise, reactants and reagents were purchased from commercial sources and used without further purification. Dry CH_2_Cl_2_, Et_2_O, THF and MeOH were obtained from a dispensing system by passing commercial material through a cartridge containing activated alumina (PURESOLV, Innovative Technology, Oldham, UK), stored under dry nitrogen, and then used as such without further drying unless specified. **DMSO** was dried by treating commercial material with CaH_2_ mesh at 150 °C under argon, followed by distillation under reduced pressure [14]. Deoxygenated and dry THF was obtained by refluxing and distilling pre-dried material (as described above) from sodium and benzophenone under argon. 

Molecular sieves were activated by heating them to 200 °C for approximately 6 h in a high vacuum and were then stored under argon [15]. 

Melting ranges were determined using a Kofler-type Leica Galen III micro hot stage microscope or an SRS OptiMelt Automated Melting Point System and are uncorrected. Temperatures are reported in intervals of 0.5 °C.

Aluminum-backed Merck silica gel 60 with the fluorescence indicator F254 was used for thin layer chromatography (TLC). Spots were visualized under UV light (254 nm) and by staining with cerium ammonium molybdate (CAM) solution (20 g of ammonium pentamolybdate, 0.8 g of cerium(IV) ammonium sulfate, 400 mL of 10 *v*/*v* % sulfuric acid) as a general purpose reagent. Alcohols were also visualized with p-anisaldehyde solution (3.5 g p-anisaldehyde, 1.5 mL acetic acid, 5 mL sulfuric acid, 120 mL ethanol), and compounds pertaining double bonds were visualized with potassium permanganate solution (1.5 g potassium permanganate, 10 g potassium carbonate, 1 mL 10 *w*/*w* % NaOH, 200 mL water).

Specific rotation was measured using an Anton Parr MCP500 polarimeter (AntonPaar GmbH, Graz, Austria) and HPLC-grade solvents under conditions as specified individually. Values are reported in the form + or—specific rotation (concentration in terms of g/100 mL, solvent). 

Analytical chromatography–spectroscopy gas chromatography–mass spectroscopy (GC-MS) was used to analyze samples of reaction products with sufficient volatility. The following instruments and columns were used: Thermo Scientific (Fisher Scientific GmbH, Schwerte, Germany) Finnigan Focus GC/Quadrupole DSQ II device using a helium flow of 2.0 mL/min, analyzing an *m*/*z* range from 50 to 650; BGB 5 (0.25 µm film; 30 m × 0.25 mm ID). Temperature gradients are as follows: Method A: 100 °C (2 min), to 280 °C in 10 min, 11 min hold-time at 280 °C (23 min); Method B: 80 °C (2 min), to 280 °C in 10 min (20 °C/min), 12 min hold-time at 280 °C (24 min); Method C: 100 °C (2 min), to 280 °C in 4.5 min (40 °C/min), 16.5 min hold-time at 280 °C (23 min); Method D: 100 °C (2 min), to 280 °C in 4.5 min (40 °C/min), 31.5 min hold-time at 280 °C (38 min); Method E: 100 °C (2 min), to 280 °C in 4.5 min (40 °C/min), 41.5 min hold-time at 280 °C (48 min). Data are reported in the form retention time; *m*/*z*1 (relative intensity in %), *m*/*z*2 (relative intensity in %), Only signals with *m*/*z* ≥ 90 and relative intensity ≥ 15% are given, except for the signal at 100% relative intensity, which is always given. Additionally, the molecular ion signal M+ is given regardless of its intensity or *m*/*z*; in cases where M+ was not visible due to excessive fragmentation, a characteristic fragment signal is identified instead. 

High-pressure liquid chromatography (HPLC) was used to determine the enantiomeric excess of reaction products, using a Dionex UltiMate 3000 device (RS Diode Array Detector Fisher Scientific GmbH, Schwerte, Germany). Chiral separation columns and analysis conditions are specified individually. In all cases, retention times include appropriate guard cartridges containing the same stationary phase as the separation column. 

Liquid chromatography–high-resolution mass spectroscopy (LC-HRMS) was used to confirm the exact molecular mass of reaction products by their quasi-molecular ions (M + H^+^ or M + Na^+^). The following two instruments were used: (1) Shimadzu Prominence HPLC device (DGU-20 A3 degassing unit, 2 × LC20AD binary gradient pump, SIL-20 A auto injector, CTO-20AC column oven, CBM-20A control module, and SPD-M20A diode array detector). Samples were eluted through a Phenomenex Kinetex precolumn (5 μm core shell ODS(3) phase; 4 mm × 2 mm ID) at 40 °C under conditions comprising gradients of H_2_O/MeOH containing formic acid (0.1 *v*/*v* %), and then detected using a Shimadzu IT-TOF-MS by electrospray ionization (ESI) or atmospheric pressure chemical ionization (APCI), as indicated individually. Analyses were performed by E. Rosenberg (CTA, VUT) and L. Czollner (IAS, VUT); (2) Agilent 1100/1200 HPLC device (degassing unit, 1200SL binary gradient pump, column thermostat, and CTC Analytics HTC PAL autosampler). Samples were eluted through a silica-based Phenomenex C-18 Security guard cartridge (1.7 µm PD; 2.1 mm ID) at 40 °C under isocratic conditions comprising H_2_O containing formic acid (0.1 *v*/*v* %)/MeOH containing formic acid (0.1 *v*/*v* %) in a ratio of 30:70 at a flow rate of 0.5 mL/min, and then detected using an Agilent 6230 LC-TOF-MS equipped with an Agilent Dual AJS ESI source by electrospray ionization (ESI). Analyses were performed by L. Czollner (IAS, VUT). Flash column chromatography was carried out on Merck silica gel 60 (40–63 μm), and separations were performed using a Büchi Sepacore system (dual Pump Module C-605, Pump Manager C-615, Fraction Collector C-660, and UV Monitor C-630 or UV Photometer C635). 

Preparative high-performance liquid chromatography (preparative HPLC) was carried out on a Phenomenex Luna reverse-phase column (10 µm C18(2) phase, 100 A; 250 mm × 21.20 mm ID), and separations were performed using a Shimadzu LC-8A device (SIL-10AP autosampler, SPD-20 detector, and FRC-10A fraction collector (Shimadzu Österreich, Korneuburg, Austria). Reaction temperatures were measured externally (electronic thermometer connected to a heater–stirrer or low-temperature thermometer in the case of cryogenic reactions), unless otherwise noted. 

Nuclear magnetic resonance spectroscopy (NMR) spectra were recorded from CDCl_3_ or DMSO-d6 solutions on a Bruker AC 200 (200 MHz proton resonance frequency) or a Bruker Advanced UltraShield (400 MHz) spectrometer (as indicated individually) from Bruker Daltonik GmbH, Bremen, Germany, and chemical shifts are reported in ascending order in ppm relative to the nominal residual solvent signals, i.e., 1H: δ = 2.50 ppm (DMSO-d6); 13C: δ = 77.16 ppm (CDCl_3_), δ = 39.52 ppm (DMSO-d6) [5,6]. For all 1H spectra in CDCl_3_, however, shifts are reported relative to tetramethyl silane (TMS) as an internal standard (δ = 0 ppm) due to the interference of aromatic signals of many samples with the residual solvent signal of CDCl_3_. For the 13C spectra, the J-modulated attached proton test (APT) or distortionless enhancement by polarization transfer (DEPT-135) pulse sequences were used to aid in the assignment. 

NF-κB transactivation activity was determined by a luciferase reporter gene assay in HEK293 cells stably transfected with the NF-κB-driven luciferase reporter gene NF-κB-luc (293/NF-κB-luc cells, Panomics, Fremont, CA, USA, RC0014) [16]. Cells were stained with 2 µM cell tracker green (CTG, Thermo Scientific). After one hour, 4 × 10⁴ cells per well were seeded in a 96-well plate in serum-free DMEM (4.5 g/L glucose) obtained from Lonza and supplemented with 2 mM glutamine, 100 U/mL benzylpenicillin and 100 µg/mL streptomycin. After incubation at 37 °C, 5% CO_2_ overnight, cells were pre-treated on the next day with test samples for 1 h. Thereafter, cells were stimulated with 2 ng/mL human recombinant TNF-α (Sigma-Aldrich Handels Gmbh, Vienna, Austria) for 4 h to activate the NF-κB signaling pathway. Then, the medium was removed, and cells were lysed with luciferase reporter lysis buffer (E3971, Promega, Madison, WI, USA). Leoligin and its derivatives were tested at a concentration of 20 µM (screening) in at least three independent experiments. The sesquiterpene lactone parthenolide, an effective inhibitor of the NF-κB pathway [12], was used as a positive control at a concentration of 5 µM and 0.1% DMSO served as vehicle control. The luminescence of the firefly luciferase product and the CTG-derived fluorescence were quantified on a Tecan GeniosPro plate reader (Tecan, Grödig, Austria). The ratio of luminescence units to fluorescence units was calculated to account for differences in cell number. Results were expressed as fold changed relative to the vehicle control with TNFα, which was set to 1 [16]. CTG-fluorescence values used to estimate cell viability were also normalized to the vehicle control with TNFα. Compared to the vehicle control, treatments with fluorescence values below 0.75 were considered as toxic.

IC_50_ values were determined by the luciferase reporter assay, as described above. Dose–response curves were established by measuring compounds at concentrations of 20 µM, 10 µM, 5 µM and 1 µM, each concentration in three independent experiments. For statistical analyses, the IC_50_ values were calculated using nonlinear regression, with sigmoidal dose responses (GraphPad Software 4.03., Inc., San Diego, CA, USA).

### 2.2. Syntheses of Target Compounds via Steglich Esterification

#### 2.2.1. ((2S,3R,4R)-4-(3,4-Dimethoxybenzyl)-2-(3,4-dimethoxyphenyl)tetrahydrofuran-3-yl)methyl cyclohex-1-enecarboxylate (3)

**Scheme 1:**
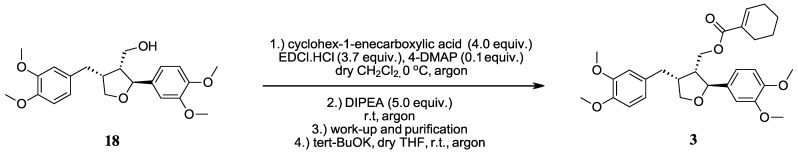
Synthesis of cyclohexenyl derivative **3**.

A reaction vessel was charged with a stirring bar, cyclohex-1-enecarboxylic acid (45.4 mg, 0.360 mmol, 4.0 equiv.) and 4-DMAP (1.1 mg, 9.0 μmol, 0.1 equiv.), and then evacuated and back-filled with argon using standard Schlenk technique. Dry CH_2_Cl_2_ (1.0 mL) was then added via syringe and the solution was cooled to 0 °C in an ice bath. The vessel was briefly opened, EDCI.HCl (63.8 mg, 0.333 mmol, 3.7 equiv.) was added in one go, and the mixture was stirred for 3 h at 0 °C. Meanwhile, a second vessel was charged with a stirring bar and starting material **18** (35.0 mg, 0.090 mmol, 1.00 equiv.), evacuated and back-filled with argon (3×), and DIPEA (78 μL, 0.45 mmol, 5.0 equiv.) was added via syringe. After 3 h, the solution containing the activated carboxylic acid was transferred to the second vial via syringe and stirred for 24 h at room temperature. The reaction solution was used directly for flash column chromatography (9 g silica, flow rate 20 mL/min, EtOAc/LP, 10:90 to 22:78 in 9 min, then 22:78 isocratically for 6 min, then to 62:38 in 30 min) to afford the title compound **3** with an overall yield of 60% as a colorless oil (Scheme 1). [α]_D_^20^: +20.5 (c 2.64, MeOH) LC-HRMS (ESI): calculated for M + Na^+^: 519.2353, found: 519.2378, Δ: 4.81 ppm. (log *P*)_calc_: 5.95 ± 0.45. GC-MS (EI, 70 eV, Method E): 48.95 min; 496.3 (M^+^, 1), 370.2 (15), 219.1 (32), 207.0 (24), 206.1 (16), 189.1 (17), 177.1 (17), 166.1 (15), 165.1 (89), 152.1 (16), 151.1 (100), 109.1 (39), 107.1 (19). ^1^H NMR (200 MHz, CDCl_3_): δ 1.51–1.71 (m, 4H), 2.10–2.27 (m, 4H), 2.50–2.95 (m, 4H), 3.76 (dd, ^2^*J* = 8.5 Hz, ^3^*J* = 6.2 Hz, 1H), 3.86 (s, 3H), 3.87 (s, 6H), 3.88 (s, 3H), 4.08 (dd, ^2^*J* = 8.5 Hz, ^3^*J* = 6.2 Hz, 1H), 4.26 (dd, ^2^*J* = 11.3 Hz, ^3^*J* = 7.1 Hz, 1), 4.42 (dd, ^2^*J* = 11.3 Hz, ^3^*J* = 6.5 Hz, 1H), 4.83 (d, ^3^*J* = 6.3 Hz, 1H), 6.67–6.92 (m, 7H). ^13^C NMR (50 MHz, CDCl_3_): δ 21.5 (d), 22.1 (d), 24.2 (d), 25.9 (d), 33.4 (t), 42.8 (d), 49.2 (d), 56.00 (q), 56.02 (q), 56.03 (q), 56.04 (q), 62.6 (t), 72.9 (t), 83.3 (d), 109.1 (d), 111.1 (d), 111.4 (d), 112.0 (d), 118.3 (d), 120.6 (d), 130.1 (s), 132.8 (s), 135.1 (s), 140.5 (d), 147.6 (s), 148.5 (s), 149.1 (s), 149.1 (s), 167.5 (s). 

#### 2.2.2. ((2S,3R,4R)-4-(3,4-Dimethoxybenzyl)-2-(3,4-dimethoxyphenyl)tetrahydrofuran-3-yl)methyl cyclobutanecarboxylate (7)

Analogous to **3**, using starting material **18** (21.5 mg, 0.055 mmol, 1.00 equiv.), cyclobutanecarboxylic acid (12.7 mg, 0.127 mmol, 2.3 equiv.), EDCI.HCl (21.2 mg, 0.111 mmol, 2.0 equiv.), 4-DMAP (0.7 mg, 5.5 µmol, 0.1 equiv.) and DIPEA (24 µL, 0.14 mmol, 2.5 equiv.), and stirring the reaction for 24 h. The reaction solution was used directly for flash column chromatography (9 g silica, flow rate 20 mL/min, EtOAc/LP, 10:90 to 15:85 in 10 min, then to 25:75 in 5 min, then to 50:50 in 7 min) to afford the title compound **7** with an overall yield of 83% as a colorless oil. [α]_D_^23^: +22.8 (c 1.27, MeOH). LC-HRMS (APCI): calculated for M + Na^+^: 493.2197, found: 493.2214, Δ: 3.45 ppm. (log *P*)_calc_: 4.62 ± 0.44. GC-MS (EI, 70 eV, Method D): 22.60 min; 470.1 (M^+^, 4), 207.0 (52), 177.1 (16), 166.1 (17), 165.1 (50), 151.0 (100), 107.1 (15). ^1^H NMR (200 MHz, CDCl_3_): δ 1.79–2.06 (m, 2H), 2.07–2.37 (m, 4H), 2.47–2.93 (m, 4H), 3.11 (quint, ^3^*J* = 8.6 Hz, 1H), 3.75 (dd, ^2^*J* = 8.5 Hz, ^3^*J* = 6.3 Hz, 1H), 3.86 (s, 3H), 3.87 (s, 6H), 3.89 (s, 3H), 4.07 (dd, ^2^*J* = 8.5 Hz, ^3^*J* = 6.3 Hz, 1H), 4.19 (dd, ^2^*J* = 11.2 Hz, ^3^*J* = 7.1 Hz, 1H), 4.38 (dd, ^2^*J* = 11.2 Hz, ^3^*J* = 6.9 Hz, 1H), 4.80 (d, ^3^*J* = 6.4 Hz, 1H), 6.66–6.90 (m, 6H). ^13^C NMR (50 MHz, CDCl_3_): δ 18.6 (t), 25.4 (t), 25.4 (t), 33.3 (t), 38.2 (d), 42.7 (d), 49.2 (d), 56.0 (q), 62.6 (t), 72.9 (t), 83.1 (d), 109.0 (d), 111.1 (d), 111.5 (d), 112.0 (d), 118.2 (d), 120.6 (d), 132.7 (s), 135.1 (s), 147.6 (s), 148.6 (s), 149.1 (s), 149.2 (s), 175.4 (s). 

#### 2.2.3. ((2S,3R,4R)-4-(3,4-Dimethoxybenzyl)-2-(3,4-dimethoxyphenyl)tetrahydrofuran-3-yl)methylbutyrate (8) 

Analogous to **3**, using starting material **18** (35.0 mg, 0.090 mmol, 1.00 equiv.), butyric acid (18.2 mg, 0.207 mmol, 2.3 equiv.), EDCI.HCl (34.5 mg, 0.180 mmol, 2.0 equiv.), 4-DMAP (1.1 mg, 9.0 µmol, 0.1 equiv.) and DIPEA (39 µL, 0.23 mmol, 2.5 equiv.). The reaction solution was used directly for flash column chromatography (9 g silica, flow rate 20 mL/min, EtOAc/LP, 10:90 to 22:78 in 9 min, then 22:78 isocratically for 6 min, then to 62:38 in 30 min) to afford the title compound **8** with an overall yield of 85% as a colorless oil. [α]_D_^20^: +20.3 (c 1.77, MeOH). LC-HRMS (ESI): calculated for M + Na^+^: 481.2197, found: 481.2207, Δ: 2.08 ppm. (log *P*)_calc_:4.75 ± 0.44. GC-MS (EI, 70 eV, Method D): 20.50 min; 458.2 (M^+^, 6), 219.1 (21), 165.1 (52), 152.1 (15), 151.1 (100). ^1^H NMR (200 MHz, CDCl_3_): δ 0.94 (t, ^3^*J* = 7.4 Hz, 3H), 1.64 (sext, ^3^*J* = 7.4 Hz, 2H), 2.26 (t, ^3^*J* = 7.4 Hz, 2H), 2.47–2.93 (m, 4H), 3.75 (dd, ^2^*J* = 8.6 Hz, ^3^*J* = 6.2 Hz, 1H), 3.86 (s, 3H), 3.87 (s, 6H), 3.89 (s, 3H), 4.07 (dd, ^2^*J* = 8.6 Hz, ^3^*J* = 6.3 Hz, 1H), 4.19 (dd, ^2^*J* = 11.2 Hz, ^3^*J* = 7.1 Hz, 1H), 4.38 (dd, ^2^*J* = 11.2 Hz, ^3^*J* = 6.9 Hz, 1H), 4.79 (d, ^3^*J* = 6.4 Hz, 1H), 6.64–6.92 (m, 6H). ^13^C NMR (50 MHz, CDCl_3_): δ 13.8 (q), 18.5 (t), 33.3 (t), 36.3 (t), 42.6 (d), 49.1 (d), 56.0 (q), 62.5 (t), 72.8 (t), 83.0 (d), 109.0 (d), 111.1 (d), 111.4 (d), 112.0 (d), 118.2 (d), 120.5 (d), 132.7 (s), 135.0 (s), 147.6 (s), 148.6 (s), 149.0 (s), 149.1 (s), 173.6 (s). 

#### 2.2.4. ((2S,3R,4R)-4-(3,4-Dimethoxybenzyl)-2-(3,4-dimethoxyphenyl)tetrahydrofuran-3-yl)methyl 3,3-dimethylbutanoate (10) 

Analogous to **3**, using starting material **18** (30.0 mg, 0.077 mmol, 1.00 equiv.) and 3,3-dimethylbutanoic acid (20.6 mg, 0.178 mmol, 2.3 equiv.), EDCI.HCl (29.6 mg, 0.154 mmol, 2.0 equiv.), 4-DMAP (0.9 mg, 7.7 µmol, 0.1 equiv.) and DIPEA (34 µL, 0.19 mmol, 2.5 equiv.). The reaction solution was used directly for flash column chromatography (9 g silica, flow rate 20 mL/min, EtOAc/LP, 10:90 to 30:70 in 30 min) to afford the title compound **10** with an overall yield of 51% as a colorless oil. [α]_D_^23^: +24.7 (c 1.01, MeOH). LC-HRMS (APCI): calculated for M+Na^+^: 509.2510, found: 509.2512, Δ: 0.39 ppm. (log *P*)_calc_: 5.45 ± 0.45. GC-MS (EI, 70 eV, Method D): 18.75 min; 486.1 (M^+^, 4), 219.0 (17), 166.0 (17), 165.0 (45), 152.1 (15), 151.0 (100). ^1^H NMR (200 MHz, CDCl_3_): δ 1.03 (s, 9H), 2.20 (s, 2H), 2.47–2.63 (m, 2H), 2.64–2.82 (m, 1H), 2.88 (dd, ^2^*J* = 12.5 Hz, ^3^*J* = 4.2 Hz, 1H), 3.76 (dd, ^2^*J* = 8.6 Hz, ^3^*J* = 6.1 Hz, 1H), 3.86 (s, 3H), 3.87 (s, 3H), 3.87 (s, 3H), 3.89 (s, 3H), 4.06 (dd, ^2^*J* = 8.6 Hz, ^3^*J* = 6.2 Hz, 1H), 4.16 (dd, ^2^*J* = 11.3 Hz, ^3^*J* = 6.9 Hz, 1H), 4.36 (dd, ^2^*J* = 11.3 Hz, ^3^*J* = 7.2 Hz, 1H), 4.80 (d, ^3^*J* = 6.4 Hz, 1H), 6.66–6.90 (m, 6H). ^13^C NMR (50 MHz, CDCl_3_): δ 29.8 (q), 30.9 (s), 33.2 (t), 42.6 (d), 48.1 (t), 49.3 (d), 56.0 (q), 62.3 (t), 72.8 (t), 82.9 (d), 109.0 (d), 111.2 (d), 111.5 (d), 112.0 (d), 118.2 (d), 120.6 (d), 132.7 (s), 135.1 (s), 147.7 (s), 148.6 (s), 149.1 (s), 149.2 (s), 172.4 (s). 

#### 2.2.5. ((2S,3R,4R)-4-(3,4-Dimethoxybenzyl)-2-(3,4-dimethoxyphenyl)tetrahydrofuran-3-yl)methyl2-ethylbutanoate (11) 

Analogous to **3**, using starting material **18** (20.3 mg, 0.052 mmol, 1.00 equiv.) and 2-ethylbutanoic acid (14.0 mg, 0.120 mmol, 2.3 equiv.), EDCI.HCl (20.0 mg, 0.105 mmol, 2.0 equiv.), 4-DMAP (0.6 mg, 5.2 µmol, 0.1 equiv.) and DIPEA (23 µL, 0.13 mmol, 2.5 equiv.). The reaction solution was used directly for flash column chromatography (9 g silica, flow rate 20 mL/min, EtOAc/LP, 10:90 to 30:70 in 30 min) to afford the title compound **11** with an overall yield of 87% as a colorless oil. [α]_D_^23^: +23.3 (c 0.88, MeOH).LC-HRMS (APCI): calculated for M + Na^+^: 509.2510, found: 509.2533, Δ: 4.52 ppm. (log *P*)_calc_: 5.63 ± 0.45. GC-MS (EI, 70 eV, Method E): 23.47 min; 486.2 (M^+^, 7), 219.1 (32), 189.1 (15), 165.1 (56), 151.0 (100). ^1^H NMR (200 MHz, CDCl_3_): δ 0.90 (t, ^3^*J* = 7.4 Hz, 6H), 1.42–1.75 (m, 4H), 2.15–2.30 (m, 1H), 2.47–2.82 (m, 3H), 2.88 (dd, ^2^*J* = 12.6 Hz, ^3^*J* = 4.0 Hz, 1H), 3.76 (dd, ^2^*J* = 8.6 Hz, ^3^*J* = 6.1 Hz, 1H), 3.87 (s, 6H), 3.87 (s, 3H), 3.89 (s, 3H), 4.06 (dd, ^2^*J* = 8.6 Hz, ^3^*J* = 6.2 Hz, 1H), 4.18 (dd, ^2^*J* = 11.3 Hz, ^3^*J* = 6.8 Hz, 1H), 4.41 (dd, ^2^*J* = 11.3 Hz, ^3^*J* = 7.1 Hz, 1H), 4.81 (d, ^3^*J* = 6.4 Hz, 1H), 6.64–6.91 (m, 6H). 

#### 2.2.6. ((2S,3R,4R)-4-(3,4-Dimethoxybenzyl)-2-(4-fluorophenyl)tetrahydrofuran-3-yl)methyl 3,3-dimethylbutanoate (14)

**Scheme 2:**
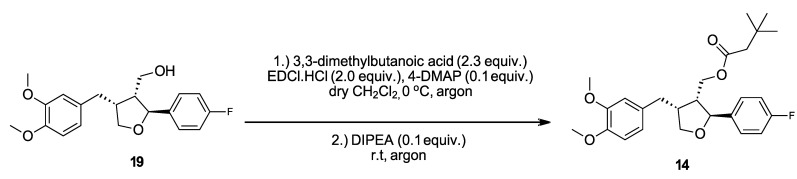
Synthesis of para-fluoro derivative **14**.

Analogous to **3**, using starting material **19** (22.0 mg, 0.064 mmol, 1.00 equiv.), 3,3-dimethylbutanoic acid (16.9 mg, 0.146 mmol, 2.3 equiv.), EDCI.HCl (24.3 mg, 0.127 mmol, 2.0 equiv.), 4-DMAP (0.8 mg, 6.4 µmol, 0.1 equiv.) and DIPEA (28 µL, 0.16 mmol, 2.5 equiv.), and stirring for 13.5 in place of 24 h. The reaction solution was used directly for flash column chromatography (9 g silica, flow rate 20 mL/min, EtOAc/LP, 9:91 to 25:75 in 60 min) to afford the title compound **14** with an overall yield of 92% as a colorless oil (Scheme 2). [α]_D_^23^: +15.7 (c 2.07, MeOH). LC-HRMS (APCI): calculated for M + H^+^: 445.2385, found: 445.2398, Δ: 2.92 ppm. (log *P*)_calc_: 5.76 ± 0.50. GC-MS (EI, 70 eV, Method D): 11.19 min; 444.1 (M^+^, 7), 194.0 (15), 190.1 (17), 189.1 (19), 177.0 (39), 164.1 (19), 152.0 (28), 151.0 (100), 123.0 (42), 109.0 (24), 107.0 (16). ^1^H NMR (200 MHz, CDCl_3_): δ 1.03 (s, 9H), 2.19 (s, 2H), 2.43–2.62 (m, 2H), 2.62–2.82 (m, 1H), 2.86 (dd, ^2^*J* = 12.5 Hz, ^3^*J* = 4.2 Hz, 1H), 3.77 (dd, ^2^*J* = 8.6 Hz, ^3^*J* = 6.3 Hz, 1H), 3.86 (s, 3H), 3.86 (s, 3H), 4.06 (dd, ^2^*J* = 8.6 Hz, ^3^*J* = 6.2 Hz, 1H), 4.16 (dd, ^2^*J* = 11.1 Hz, ^3^*J* = 7.2 Hz, 1H), 4.37 (dd, ^2^*J* = 11.3 Hz, ^3^*J* = 6.9 Hz, 1H), 4.85 (d, ^3^*J* = 6.1 Hz, 1H), 6.64–6.84 (m, 3H), 6.96–7.09 (m, 2H), 7.23–7.34 (m, 2H). ^13^C NMR (50 MHz, CDCl_3_): δ 29.8 (q), 30.9 (s), 33.1 (t), 42.6 (d), 48.1 (t), 49.5 (d), 56.00 (q), 56.04 (q), 62.2 (t), 72.9 (t), 82.7 (d), 111.5 (d), 112.0 (d), 115.4 (dd), 120.6 (d), 127.5 (dd), 132.6 (s), 138.5 (d), 147.7 (s), 149.1 (s), 162.4 (d), 172.4 (s).

#### 2.2.7. ((2S,3R,4R)-4-(3,4-Dimethoxybenzyl)-2-(4-fluorophenyl)tetrahydrofuran-3-yl)methyl cyclohexanecarboxylate (17)

Analogous to **3**, using starting material **19** (31.2 mg, 0.090 mmol, 1.00 equiv.), cyclohexanecarboxylic acid (26.5 mg, 0.207 mmol, 2.3 equiv.), EDCI.HCl (34.5 mg, 0.180 mmol, 2.0 equiv.), 4-DMAP (1.1 mg, 9.0 µmol, 0.1 equiv.) and DIPEA (39 µL, 0.23 mmol, 2.5 equiv.). The reaction solution was used directly for flash column chromatography (9 g silica, flow rate 20 mL/min, EtOAc/LP, 0:100 to 15:85 in 16 min, then to 22:78 in 4 min) to afford the title compound **17** with an overall yield of 91% as a colorless oil. [α]_D_^20^: +15.1 (c 2.10, MeOH). LC-HRMS (ESI): calculated for M+Na^+^: 479.2204, found: 479.2185, Δ: −3.96 ppm. (log *P*)_calc_: 6.06 ± 0.49. GC-MS (EI, 70 eV, Method C): 19.53 min; 456.3 (M^+^, 4), 194.1 (24), 190.1 (24), 189.1 (23), 177.1 (44), 164.1 (26), 163.1 (16), 152.1 (30), 151.1 (100), 123.0 (47), 109.1 (33), 107.1 (18). ^1^H NMR (200 MHz, CDCl_3_): δ 1.11–1.95 (m, 10H), 2.17–2.34 (m, 1H), 2.42–2.62 (m, 2H), 2.62–2.79 (m, 1H), 2.85 (dd, ^2^*J* = 12.4 Hz, ^3^*J* = 4.3 Hz, 1H), 3.77 (dd, ^2^*J* = 8.6 Hz, ^3^*J* = 6.3 Hz, 1H), 3.86 (s, 3H), 3.87 (s, 3H), 4.07 (dd, ^2^*J* = 8.6 Hz, ^3^*J* = 6.3 Hz, 1H), 4.17 (dd, ^2^*J* = 11.3 Hz, ^3^*J* = 7.3 Hz, 1H), 4.38 (dd, ^2^*J* = 11.3 Hz, ^3^*J* = 6.7 Hz, 1H), 4.84 (d, ^3^*J* = 6.2 Hz, 1H), 6.65–6.75 (m, 2H), 6.81 (d, ^3^*J* = 7.9 Hz, 1H), 7.02 (dd, ^3^*J* = 8.7 Hz, ^3^*J*_H-F_ = 8.7 Hz, 2H), 7.29 (dd, ^3^*J* = 8.6 Hz, ^4^*J*_H-F_ = 5.4 Hz, 2H). ^13^C NMR (50 MHz, CDCl_3_): δ 25.5 (t), 25.8 (t), 29.1 (t), 29.1 (t), 33.2 (t), 42.7 (d), 43.3 (d), 49.5 (d), 55.97 (q), 56.00 (q), 62.3 (t), 72.9 (t), 82.7 (d), 111.4 (d), 111.9 (d), 115.4 (dd), 120.5 (d), 127.5 (dd), 132.6 (s), 138.5 (d), 147.7 (s), 149.1 (s), 162.3 (d), 176.0 (s).

## 3. Results

Molecules that could be used as NF-κB-inhibiting tool compounds for further investigation should possess an inhibitory potency comparable to that of parthenolide [17,18,19,20], a sesquiterpene lactone from feverfew *Tanacetum parthenium*, which modulates the NF-κB-mediated inflammatory response, e.g., in experimental atherosclerosis [21], and induces apoptosis in acute myelogenous leukemia (AML) cells [22,23]. Therefore, parthenolide was used as a positive control for comparing NF-κB IC_50_ values of the synthetic lignan library. The IC_50_ value of parthenolide in NF-κB inhibition was determined to be 1.7 µM in our assay system. The IC_50_ of leoligin itself is around 20 µM [5]. For the purpose of this study, compounds which did not exert appreciable activity (no more than 50% inhibition at a single-dose concentration of 20 µM) were not considered for more detailed investigation. Thus, we screened a subset of synthetic analogs for NF-κB inhibition, modified at two positions, either at the ester functionality or at the aryl group in position 2. 

**Scheme 3:**
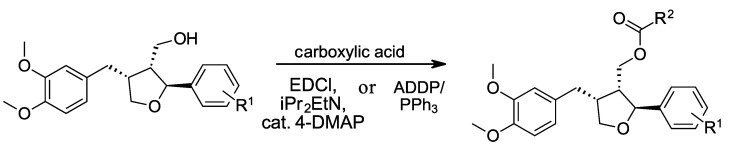
General scheme for Steglich and Mitsunobu esterification.

The complete total synthesis of leoligin has previously been described by our group [5]. In this paper, it was hypothesized that ester functionalities which possess Michael-acceptor properties are detrimental for high NF-κB inhibition properties. However, this hypothesis was based only on the assessment of leoligin itself and four additional ester derivatives. Hence, the evaluation of more ester derivatives has become mandatory, in order to prove or disprove this theory. Consequently, a set of compounds with different ester groups was synthesized (experimental and analytical details of synthesized compounds can be found in the supporting information, Supplementary Materials). Various saturated, unsaturated and aromatic carboxylic acids were employed to carry out the esterification following either a Steglich or Mitsunobu protocol [5]. Several α,β-unsaturated carboxylic acids underwent successful Steglich esterification under mild conditions. However, leoligin had to be prepared by following a Mitsunobu protocol to avoid the double-bond isomerization in the ester moiety. This was otherwise likely under typical Steglich conditions due to EDCl-mediated (*N*-(3-dimethylaminopropyl)-*N′*-ethylcarbodiimide hydrochloride) acid activation and reversible Michael addition by the 4-dimethylaminophenol (4-DMAP) acylation catalyst, resulting in bond rotation to the thermodynamically more stable (*E*) configuration (Scheme 3).

Thus, to preserve the integrity of the Z-configuration of the α,β-unsaturated system during esterification, a Mitsunobu protocol was applied for the generation of leoligin (and generally, of angelic acid-bearing lignans), using diethyl azodicarboxylate (DEAD) as the reagent. As a side note, DEAD can be replaced by 1,1′-(azodicarbonyl)dipiperidine (ADDP) in order to simplify purification by column chromatography in some cases, because its reduction byproduct possesses significantly different polarity as compared with the target products [24,25]. Overall, 17 different leoligin derivatives were prepared, whereas our group has previously described the synthesis of some compounds reported here in the literature, but in a different context. For instance, leoligin and compounds **1**, **9** and **12** have already been investigated regarding NF-κB and VSMC proliferation inhibition [5], and the IC_50_ values for those compounds are reproduced in the present manuscript. 5-Methoxyleoligin, derivatives **4** and **5**, have been explored as potential cholesterol efflux promoters [4], whereas compounds **2** and **4** have been characterized as farnesoid X receptor agonists and modulators of cholesterol transport [26]. Last but not least, compounds **6**, **13**, **15** and **16** have been included in a patent application [27].

**Figure 2:**
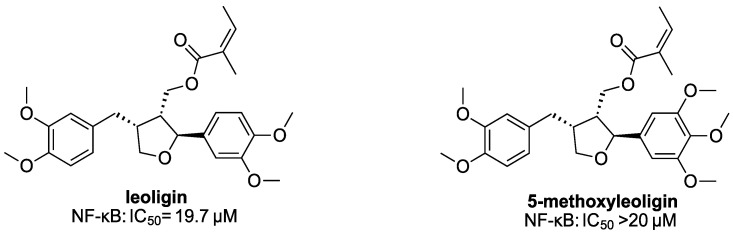
Structures of leoligin and 5-methoxyleoligin.

The synthesized ester derivatives were screened in a first round at a single dose of 20 µM. IC_50_ values of promising analogs were determined by dose–response measurements at 1 to 20 µM. Higher concentrations were not tested, because only compounds with IC_50_ values lower than that of the natural occurring leoligin were of interest. The parent compounds leoligin and 5-methoxyleoligin showed only a very moderate inhibitory effect on NF-κB, with 19.7 μΜ and >20 μΜ, respectively (Figure 2). 

According to our previous report [5], the initial hypothesis was that leoligin and 5-methoxyleoligin might very well inhibit the NF-κB pathway more effectively, if it were not for the angelic acid ester providing a point of attack in the metabolism of the cell because it is a good Michael acceptor in which addition to the double bond system can easily occur with a range of nucleophiles. For this purpose, esters **1**, **2** and **3** were evaluated (Table 1). Indeed, we observed significantly higher NF-κB inhibition for all three derivatives, with IC_50_ values of 5.3 µM for **1** (data already reported in [5]), 6.5 µM for **2** and 4.9 µM for **3**, respectively. Compound **1** is likely least prone to Michael addition due to steric shielding of the β-carbon in the α,β-unsaturated system, but this position should also be less reactive in compounds **2** and **3**, because embedding the double bond in a cyclic system increases its stability. To further test this hypothesis, compound **4** was prepared, which carries a phenyl ring in the ester moiety and is not a Michael acceptor at all. Interestingly, no inhibitory activity at 20 µM concentration was observed in this case (Table 1).

The question arose as to whether the extended π-system in **4** is detrimental for the desired activity and whether a π-system is required at all. Therefore, we tested compound **5** as the saturated analog of **4** where the phenyl ring is replaced by a cyclohexyl ring. The result was remarkable, because **5** gave an IC_50_ value as low as 3.2 µM, indicating that the π-system in **4** is indeed detrimental (Table 1). Decreasing the ring size to cyclopentyl (compound **6**, IC_50_ 8.0 µM) and cyclobutyl (compound **7**, IC_50_ 6.4 µM) still gave active compounds with somewhat higher IC_50_ values (Table 1). Additionally, an open chain derivative, *n*-propyl derivative **8**, was tested as well and showed inhibitory activity, with an IC_50_ value of 6.3 μM, demonstrating that neither a π-system nor a cyclic system is necessarily required for NF-κB inhibition. 

Out of the derivatives investigated thus far, cyclohexylester **5** is the most sterically demanding. Hence, in the next step we investigated how much steric bulk is tolerated in the ester position and whether even larger groups could have a beneficial effect. The *t*-butyl group was a natural choice to test for steric effects, and in fact, compound **9** exhibited a very low IC_50_ of 2.2 μM (Table 1). Similarly, 3,3-dimethylbutanoate **10** also showed significant NF-κB inhibition with an IC_50_ of 4.7 μM as well as 3-pentyl derivative **11** (IC_50_: 4.9 μM). However, the lowest IC_50_ of 1.6 μM we observed in this study was found when we installed the cycloheptyl ring in compound **12** (Table 1). At this point, we could conclude that aliphatic esters of substantial size are required to obtain a strong inhibitory effect on NF-κB activation.

Next, we investigated whether modifications to the substitution pattern on the C2-aryl ring influence NF-κB inhibition. First, 5-methoxyleoligin analog **13** (akin to compound **8** from the leoligin series, Table 1) was prepared in order to see whether aliphatic esters have a beneficial effect on this scaffold as well. However, in this case, no inhibitory activity was observed at 20 μM. Consequently, no further derivatives of this series were investigated.

In a previous study, we had already investigated modifications to the aryl ring at position 2. Therein, an activity profile in favor of VSMC proliferation inhibition without cytotoxicity was observed when substituting the 3,4-dimethoxy groups with *p*-fluorine [5]. NF-κB inhibition was rather weak, but for these derivatives, we still relied on the angelic acid ester which was shown to be detrimental for NF-κB inhibitory activity in the present study. Hence, we prepared *p*-fluorine derivatives with different ester functionalities and subjected them to our test assay. Table 1 compares the observed activities of the corresponding 3,4-dimethoxy ester variations with their 4-fluoro counterparts. In this comparison, similar potencies were observed for cyclic and acyclic aliphatic esters. For instance, compounds **14** and **10** (bearing the 3,3-dimethylbutanoate) exhibited very similar NF-κB inhibition (IC_50_ of 3.7 μΜ and 4.7 μΜ, respectively). The same was true for compounds **15** and **11** (IC_50_ of 5.3 μΜ and 4.9 μΜ, respectively) and all other pairs of compounds (IC_50_ of 3.9 μΜ for **16** vs. 8.0 μΜ for **6,** and 6.0 μΜ for **17** vs. 3.2 μΜ for **5**). 

## 4. Conclusions

In conclusion, we could show that modifications to the ester functionality have a tremendous impact on the inhibition of NF-κB activation. Metabolic stability of the ester group plays an important role and steric bulk is well tolerated and even required in this position. On the other hand, a π-system, either in the form of a phenyl ring or an isolated double bond, is not required to obtain the desired activity. Additionally, the substituents on the aryl ring in C2-position have an effect because an additional methoxy group was detrimental, but a single fluoro substituent in *para*-position led to similar activity, again with the proper ester in place. Overall, we succeeded in revealing the structure–activity relationship of leoligin-derived derivatives in the context of NF-κB inhibition, paving the way for further structural refinements, e.g., via investigating further changes to the C2-aryl moiety as well as to the C4-aryl ring.

## Figures and Tables

**Table 1:** Structures and IC_50_ values of tested leologin derivatives.

Entry	Structure/No	IC_50_/μM
1	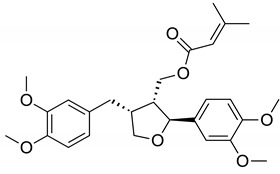 **1**	5.3
2	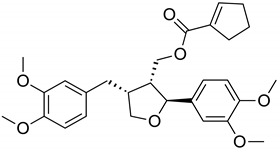 **2**	6.5
3	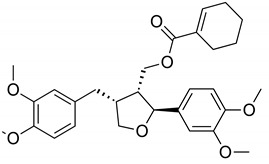 **3**	4.9
4	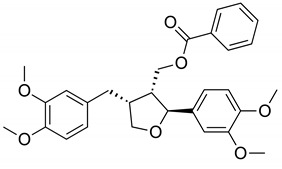 **4**	>20
5	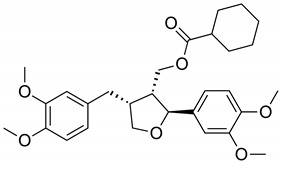 **5**	3.2
6	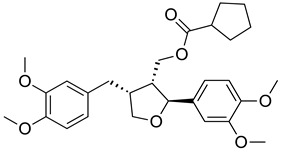 **6**	8.0
7	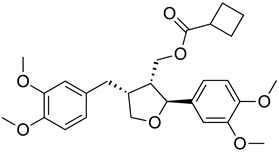 **7**	6.4
8	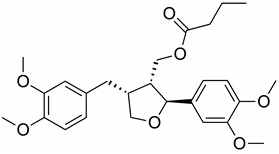 **8**	6.3
9	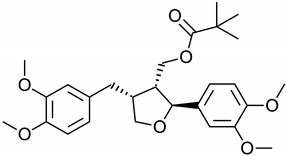 **9**	2.2
10	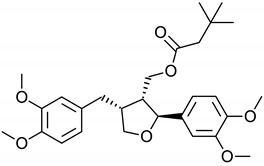 **10**	4.7
11	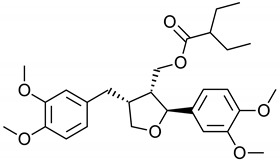 **11**	4.9
12	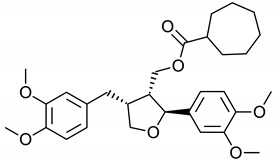 **12**	1.6
13	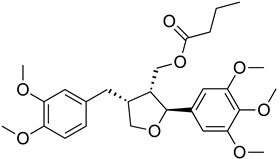 **13**	>20
14	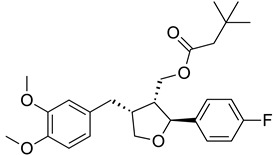 **14**	3.7
15	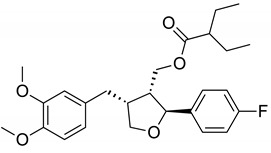 **15**	5.3
16	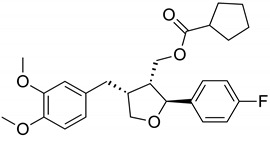 **16**	3.9
17	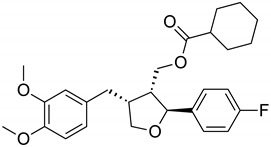 **17**	6.0

## Data Availability

The data that support the findings of this study are available from the corresponding author upon reasonable request.

## Supplementary Materials

The following supporting information can be downloaded at: https://www.mdpi.com/article/10.3390/biomedicines10010062/s1, Experimental details for all prepared compounds including analytical data.
