# Supplementary material for: Investigation of Leoligin Derivatives as NF-κΒ Inhibitory Agents
Source: Biomedicines. 2021 Dec 28;10(1):62. doi: 10.3390/biomedicines10010062 (PMC8773117; doi:10.3390/biomedicines10010062)
Supplement: Supplementary file 1 [file biomedicines-10-00062-s001.zip › biomedicines-1453998-supplementary.pdf]

Supporting Information

Investigation of Leoligin Derivatives as NF-κB Inhibitory Agents

Thomas Linder,[a] Eleni Papaplioura,[a] Diyana Ogurlu,[b] Sophie Geyrhofer,[a] Scarlet Hummelbrunner,[b] Daniel Schachner,[b] Atanas G. Atanasov,[b,c,d] Marko D. Mihovilovic,[a] Verena M. Dirsch,[b,*] Michael Schnürch[a,*]

a) TU Wien, Institute for Applied Synthetic Chemistry, Getreidemarkt 9/163, 1060 Vienna, Austria

b) Department of Pharmaceutical Sciences, University of Vienna, Althanstraße 14, 1090 Vienna, Austria

c) Ludwig Boltzmann Institute for Digital Health and Patient Safety, Medical University of Vienna, Spitalgasse 23, 1090 Vienna, Austria

d) Institute of Genetics and Animal Biotechnology of the Polish Academy of Sciences, Jastrzebiec, 05-552 Magdalenka, Poland

verena.dirsch@univie.ac.at, michael.schnuerch@tuwien.ac.at

General Notes

Chemicals

Unless noted otherwise, reactants and reagents were purchased from commercial sources and used without further purification.

Dry CH_2Cl_2, Et_2O, THF and MeOH were obtained from a dispensing system by passing commercial material through a cartridge containing activated alumina (PURESOLV, Innovative Technology), stored under dry nitrogen and then used as such without further drying unless specified.

DMSO was dried by treating commercial material with CaH2 mesh at 150 °C under argon, followed by distillation under reduced pressure. [1]

Deoxygenated and dry THF was obtained by refluxing and distilling pre-dried material (as described above) from sodium and benzophenone under argon.

Molecular sieves were activated by heating them to 200 °C for approximately 6 h in high vacuum and were then stored under argon. [2]

Melting ranges were determined using a Kofler-type Leica Galen III micro hot stage microscope or an SRS OptiMelt Automated Melting Point System and are uncorrected. Temperatures are reported in intervals of 0.5 °C.

Aluminum-backed Merck silica gel 60 with fluorescence indicator F254 was used for **Thin Layer Chromatography** (TLC). Spots were visualized under UV light (254 nm) and by staining with cerium ammonium molybdate (CAM) solution (20 g of ammonium pentamolybdate, 0.8 g of cerium(IV) ammonium sulfate, 400 mL of 10 v/v % sulfuric acid) as a general purpose reagent. Alcohols were also visualized with p-anisaldehyde solution (3.5 g p-anisaldehyde, 1.5 mL acetic acid, 5 mL sulfuric acid, 120 mL ethanol), and compounds pertaining double bonds were visualized with potassium permangante solution (1.5 g potassium permanganate, 10 g potassium carbonate, 1 mL 10 w/w % NaOH, 200 mL water).

Specific rotation was measured using an Anton Parr MCP500 polarimeter and HPLC grade solvents under conditions as specified individually. Values are reported in the form + or - specific rotation (concentration in terms of g / 100 mL, solvent).

Analytical Chromatography-Spectroscopy

Gas Chromatography-Mass Spectroscopy (GC-MS) was used to analyze samples of reaction products with sufficient volatility. The following instruments and columns were used:

Instrument: Thermo Scientific Finnigan Focus GC / Quadrupole DSQ II device using a helium flow of 2.0 mL / min, analyzing an m/z range from 50 to 650.

Column: BGB 5 (0.25 µm film; 30 m x 0.25 mm ID)

Temperature gradients are as follows:

Method A: 100 °C (2 min), to 280 °C in 10 min, 11 min hold-time at 280 °C (23 min)

Method B: 80 °C (2 min), to 280 °C in 10 min (20 °C / min), 12 min hold-time at 280 °C (24 min)

Method C: 100 °C (2 min), to 280 °C in 4.5 min (40 °C / min), 16.5 min hold-time at 280 °C (23 min)

Method D: 100 °C (2 min), to 280 °C in 4.5 min (40 °C / min), 31.5 min hold-time at 280 °C (38 min)

Method E: 100 °C (2 min), to 280 °C in 4.5 min (40 °C / min), 41.5 min hold-time at 280 °C (48 min)

Data is reported in the form retention time; m/z1 (relative intensity in %), m/z2 (relative intensity in %), … Only signals with m/z ≥ 90 and relative intensity ≥ 15 % are given, except for the signal at 100 % relative intensity which is always given. Also, the molecular ion signal M+ is given regardless of its intensity or m/z; in cases where M+ was not visible due to excessive fragmentation, a characteristic fragment signal is identified instead.

High Pressure Liquid Chromatography (HPLC) was used to determine enantiomeric excess of reaction products, using a Dionex UltiMate 3000 device (RS Diode Array Detector). Chiral separation columns and analysis conditions are specified individually. In all cases, retention times include appropriate guard cartridges containing the same stationary phase as the separation column.

Liquid Chromatography-High Resolution Mass Spectroscopy (LC-HRMS) was used to confirm exact molecular mass of reaction products by their quasi-molecular ions (M+H+ or M+Na+). The following two instruments were used:

Instrument 1: Shimadzu Prominence HPLC device (DGU-20 A3 degassing unit, 2 x LC20AD binary gradient pump, SIL-20 A auto injector, CTO-20AC column oven, CBM-20A control module, and SPD-M20A diode array detector). Samples were eluted through a Phenomenex Kinetex precolumn (5 µm core shell ODS(3) phase; 4 mm x 2 mm ID) at 40 °C under conditions comprising gradients of H2O / MeOH containing formic acid (0.1 v/v %), and then detected using a Shimadzu IT-TOF-MS by Electrospray Ionization (ESI) or Atmospheric Pressure Chemical Ionization (APCI), as indicated individually. Analyses were performed by E. Rosenberg (CTA, VUT) and L. Czollner (IAS, VUT).

Instrument 2: Agilent 1100/1200 HPLC device (degassing unit, 1200SL binary gradient pump, column thermostat, and CTC Analytics HTC PAL autosampler). Samples were eluted through a silica-based Phenomenex C-18 Security guard cartridge (1.7 µm PD; 2.1 mm ID) at 40 °C under isocratic conditions comprising H2O containing formic acid (0.1 v/v %) / MeOH containing formic acid (0.1 v/v %) in a ratio of 30 : 70 at a flow rate of 0.5 mL / min, and then detected using an Agilent 6230 LC-TOF-MS equipped with an Agilent Dual AJS ESI source by Electrospray Ionization (ESI). Analyses were performed by L. Czollner (IAS, VUT).

Preparative chromatography

Flash column chromatography was carried out on Merck silica gel 60 (40-63 µm), and separations were performed using a Büchi Sepacore system (dual Pump Module C-605, Pump Manager C-615, Fraction Collector C-660, and UV Monitor C-630 or UV Photometer C635).

Preparative High Peformance Liquid Chromatography (preparative HPLC) was carried out on a Phenomenex Luna reversed-phase column (10 µm C18(2) phase, 100 A; 250 mm x 21.20 mm ID), and separations were performed using a Shimadzu LC-8A device (SIL-10AP autosampler, SPD-20 detector, and FRC-10A fraction collector).

Reaction temperatures were measured externally (electronic thermometer connected to heater-stirrer or low temperature thermometer in case of cryogenic reactions) unless otherwise noted.

Nuclear Magnetic Resonance (NMR) spectroscopy

NMR spectra were recorded from CDCl3 or DMSO-d6 solutions on a Bruker AC 200 (200 MHz proton resonance frequency) or a Bruker Advanced UltraShield (400 MHz) spectrometer (as indicated individually), and chemical shifts are reported in ascending order in ppm relative to the nominal residual solvent signals, i.e. 1H: δ = 2.50 ppm (DMSO-d6); 13C: δ = 77.16 ppm (CDCl3), δ = 39.52 ppm (DMSO-d6).[5, 6] For all 1H spectra in CDCl3, however, shifts are reported relative to TMS as internal standard (δ = 0 ppm) due to the interference of aromatic signals of many samples with the residual solvent signal of CDCl3. For 13C spectra, J-modulated (APT) or DEPT-135 pulse sequences were used to aid in the assignment.

NF-κB Reporter Gene Assay

HEK293 cells stably transfected with the NF-κB-driven luciferase reporter gene NF-κB-luc (293/NF-κB-luc cells, Panomics, RC0014) were stained with 2 µM cell tracker green (CTG, Thermo Scientific). After one hour, 4×10^4 cells per well were seeded in a 96 well plate in serum-free DMEM (4.5 g/L glucose) obtained from Lonza and supplemented with 2 mM glutamine, 100 U/mL benzylpenicillin and 100 µg/mL streptomycin. After incubation at 37 °C, 5% CO_2 overnight, the cells were pre-treated on the next day with the samples for 1 h. Thereafter, cells were stimulated with 2 ng/mL human recombinant TNF-α (Sigma) for 4 h to activate the NF-κB signaling pathway. Then the medium was removed and cells were lysed with luciferase reporter lysis buffer (E3971, Promega, Madison, USA). Leoligin and its derivatives were tested at a concentration of 20 µM (screening) or at 20 µM, 10 µM, 5 µM, 1 µM (IC_{50}) in at least three independent experiments. The sesquiterpene lactone parthenolide, an effective inhibitor of the NF-κB pathway [3], was used as a positive control at a concentration of 5 µM and 0.1% DMSO served as vehicle control. The luminescence of the firefly luciferase product and the CTG-derived fluorescence were quantified on a Tecan GeniosPro plate reader (Tecan, Austria). The ratio of luminescence units to fluorescence units was calculated to account for differences in cell number. Results were expressed as fold changed relative to the vehicle control with TNFα, which was set to 1 [4]. CTG-fluorescence values used to estimate cell viability were also normalized to the vehicle control with TNFα. Compared to the vehicle control, treatments with fluorescence values below 0.75 were considered as toxic.

Synthesis of leoligin analogs

(*Z*)-((2*S*,3*R*,4*R*)-4-(3,4-Dimethoxybenzyl)-2-(3,4-dimethoxyphenyl)tetrahydrofuran-3-yl)methyl-2-methylbut-2-enoate (**leoligin**)

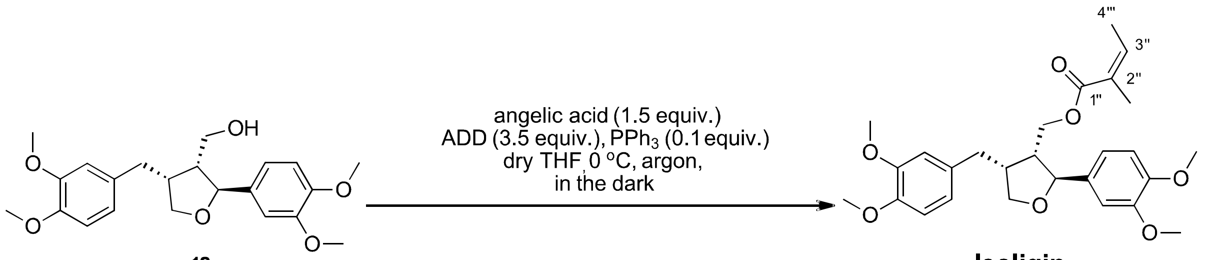

18 **leoligin**

A reaction vessel was charged with a stirring bar, starting material **18** (989 mg, 2.55 mmol, 1.0 equiv.), angelic acid (383 mg, 3.83 mmol, 1.5 equiv.) and PPh_3 (2.34 g, 8.93 mmol, 3.5 equiv.), and then evacuated and backfilled with argon using standard Schlenk technique. Dry THF (20 mL) was then added, and the solution cooled to 0 °C in an ice bath. To the stirred mixture was then added DEAD (1.40 mL, 8.93 mmol, 3.5 equiv.) dropwise *via* syringe, and the reaction stirred for 12 h while being kept away from light and allowed to warm slowly to room temperature. The solvent was evaporated, which was followed by the addition of $CHCl_3$ (15 mL), LP (300 mL) and water (200 mL). The layers were separated, and the aqueous phase was re-extracted with LP (4 x 50 ml). The solvents were evaporated from the combined organic phases and then flash column chromatography was performed (180 g silica, flow rate 40 mL / min, EtOAc / LP, 25 : 75 to 50 : 50 in 60 min) to afford the title compound **leoligin**. An analytical sample could be crystallized from a saturated solution of heptane and cooling it to -20 °C for several days.

Yield: 1.124 g, 94 %

Appearance: nearly colorless oil

Melting range: 45.0 – 46.5 °C; lit. **melting range**: n/a (natural compound obtained as a colorless amorphous substance)

***R*$_f$ (silica)**: 0.57 (EtOAc / LP, 1 : 1)

$[\alpha]_D^{25}$: +23.4 (c 3.69, MeOH); lit.**$[\alpha]_D^{20}$**: +25 (c 0.002, CH_2Cl_2)

LC-HRMS (ESI): calculated for $M+Na^+$: 493.2197, found: 493.2201, Δ: 0.81 ppm

(log *P*)$_{calc}$: 5.38 ± 0.48

GC-MS (EI, 70 eV, Method D): 23.65 min; 470.2 (M^+, 3), 219.1 (26), 189.1 (15), 177.1 (15), 165.1 (72), 151.0 (100), 107.1 (15).

^{1}H NMR (200 MHz, $CDCl_3$): δ 1.85 – 1.90 (m, 3H, H5'''), 2.00 (dq, ^3J = 7.2 Hz, ^5J = 1.5 Hz, 3H, H4'''), 2.49 – 2.85 (m, 3H, H3, H4, C4-CH), 2.90 (dd, ^2J = 12.4 Hz, ^3J = 4.2 Hz, 1H, C4-CH), 3.78 (dd, ^2J = 8.6 Hz, ^3J = 6.0 Hz, 1H, H5), 3.86 (s, 3H, Ar-OCH_3), 3.87 (s, 6H, Ar-OCH_3), 3.88 (s, 3H, Ar-OCH_3), 4.08 (dd, ^2J = 8.6 Hz, ^3J = 6.2 Hz, 1H, H5), 4.28 (dd, ^2J = 11.3 Hz, ^3J = 7.0 Hz, 1H, C3-CH), 4.42 (dd, ^2J = 11.3 Hz, ^3J = 7.0 Hz, 1H, C3-CH), 4.84 (d, ^3J = 6.3 Hz, 1H, H2), 6.10 (qq, ^3J = 7.2 Hz, ^4J = 1.3 Hz, 1H, H3'''), 6.67 – 6.75 (m, 2H, H2'', H6''), 6.77 – 6.90 (m, 4H, H2', H5', H6', H5'').

^{13}C NMR (100 MHz, $CDCl_3$): δ 16.0 (q, C4'''), 20.7 (q, C5'''), 33.3 (t, C4-C), 42.8 (d, C4), 49.3 (d, C3), 56.0 (q, 2 x Ar-OCH_3), 56.0 (q, Ar-OCH_3), 56.1 (q, Ar-OCH_3), 62.3 (t, C3-C), 72.8 (t, C5), 83.0 (d, C2), 109.0 (d, C2'), 111.2 (d, C5'), 111.4 (d, C5''*), 112.0 (d, C2''*), 118.2 (d, C6'), 120.6 (d, C6''), 127.5 (s, C2'''), 132.7 (s, C1''), 135.1 (s, C1'), 139.0 (d, C3'''), 147.6 (s, C4''), 148.6 (s, C4'), 149.1 (s, C3''), 149.2 (s, C3'), 167.8 (s, C1''').

(Z)-((2S,3R,4S)-4-(3,4-Dimethoxyphenyl)-2-(3,4,5-trimethoxyphenyl)tetrahydrofuran-3-yl)methyl 2-methylbut-2-enoate (**5-methoxyleoligin**)

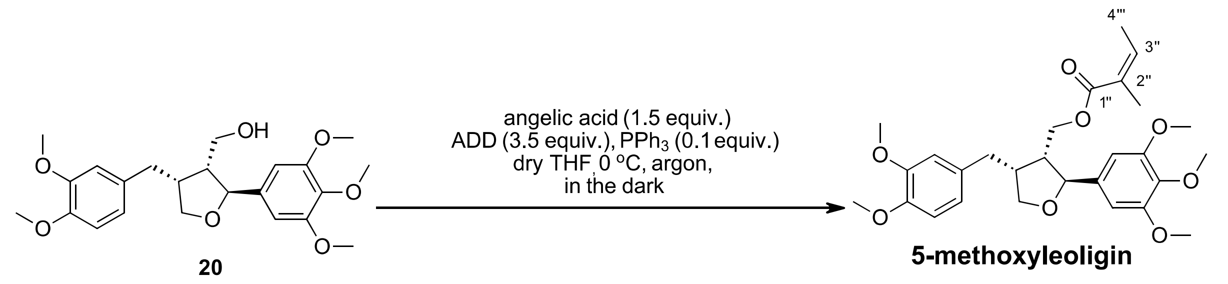

The substrate **20** (152.6 mg, 0.365 mmol), angelic acid (1.5 equiv.) and PPh3 (3.5 equiv.) were charged under argon, cooled with an ice bath and dissolved in THF (0.13 M). ADD (3.5 equiv.) was added slowly and the reaction was stirred for 18.5 h in the dark while being allowed to warm to rt. Reaction progress was monitored by TLC. When the reaction was finished, brine was added, the layers separated, and the aqueous phase was extracted with 3 x Et2O. The combined organic layers were dried over Na2SO4, filtered and the solvent was removed via evaporation. The crude product was purified via column chromatography (MPLC, 40 g silica, 50 mL/min flow rate, 10-22 % EtOAc in 10 min, then 22 % EtOAc for 10 min, then 22-65 % EtOAc in 40 min.

Yield: 134.2 mg (74 %)

Appearance: slightly yellow viscous oil

***R*f (silica)**: 0.25 (EtOAc / LP, 1 : 2)

$[\alpha]_D^{25}$: +19.7 (c 1.5415, MeOH); lit.**$[\alpha]_D^{20}$**: +20.86 (c 0.302, MeOH)

LC-HRMS (ESI): calculated for $M+Na^+$: 523.2302, found: 523.2311, Δ: 1.72 ppm

GC-MS (EI, 70 eV, Method B): 500 (M+ , 12), 249 (24), 203 (13), 196 (11), 195 (61), 190 (11), 189 (15), 181 (23), 178 (11), 177 (14), 152 (15), 151 (100), 107 (14).

^{1}H-NMR (200 MHz, CDCl3): δ 1.83-1.90 (m, 3H, α-CH3), 1.95-2.04 (m, 3H, β-CH3), 2.47- 2.81 (m, 3H, H3 & H4 & CH2), 2.88 (dd, 1H, CH2), 3.77 (dd, J= 8.70 & 6.26 Hz, 1H, H5), 3.81 (s, 3H, OCH3), 3.84 (s, 6H, 2xOCH3), 3.85 (s, 6H, 2xOCH3), 4.07 (dd, J=8.70 & 6.07 Hz, 1H, H5), 4.29 (dd, J=11.35 & 7.14, 1H, CH2O), 4.43 (dd, J=11.35 & 6.56 Hz, 1H, CH2O), 4.83 (d, J=5.87, 1H, H2), 6.02-6.17 (m, 1H, β-CH), 6.54 (s, 2H, H2' & H6'), 6.64-6.83 (m, 3H, H2'' & H5'' & H6'').

^{13}C-NMR (50 MHz, CDCl3): δ 15.8 (q, β-CH3), 20.5 (q, α-CH3), 33.12 (t, CH2), 42.56 (d, C4), 49.2 (d, C3), 55.8 (q, OCH3), 55.9 (q, OCH3), 56.1 (q, C3' OCH3 & C5' OCH3), 60.8 (q, C4' OCH3), 62.2 (t, CH2O), 72.8 (t, C5), 83.1 (d, C2), 102.5 (d, C2' & C6'), 111.3 (d, C2''), 111.8 (d, C5''), 120.4 (d, C6''), 127.2 (s, α-C), 132.7 (s, C1''), 138.2 (s, Cq), 139.0 (d, β-CH), 147.5 (s, C4''), 148.9 (s, C3''), 153.3 (s, C3' & C5'), 167.6 (s, C=O). One Cq not visible.

General Procedure for Steglich Esterification

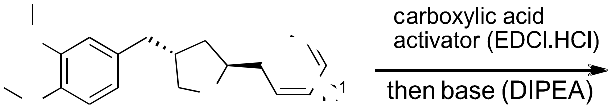

X

All compounds of generic structure **X** in this section were prepared according to the *General Outline* above and are arranged such that compounds with the same R[1] are grouped together. Thus, for the Preparation of any particular compound, the experimental details are either given in full in the analogous procedure below. Generally, reaction progress was monitored by TLC or GC-MS, and the reaction was terminated when complete. Details for work-up and purification are given for each case individually to afford compounds of structure **X**.

((2*S*,3*R*,4*R*)-4-(3,4-Dimethoxybenzyl)-2-(3,4-dimethoxyphenyl)tetrahydrofuran-3-yl)methyl 3-methylbut-2-enoate (**1**)

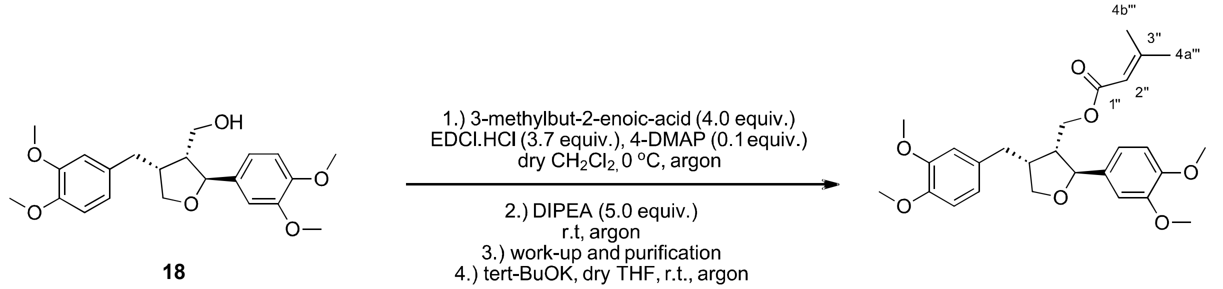

A reaction vessel was charged with a stirring bar, 3-methylbut-2-enoic acid (36.0 mg, 0.360 mmol, 4.0 equiv.) and 4-DMAP (1.1 mg, 9.0 µmol, 0.1 equiv.), and then evacuated and back-filled with argon using standard Schlenk technique (1 x). Dry CH_2Cl_2 (1.0 mL) was then added *via* syringe and the solution was cooled to 0 °C in an ice bath. The vessel was briefly opened, EDCI.HCl (63.8 mg, 0.333 mmol, 3.7 equiv.) added in one go and the mixture was stirred for 3 h at 0 °C. Meanwhile, a second vessel was charged with a stirring bar and starting material **18** (35.0 mg, 0.090 mmol, 1.00 equiv.), evacuated and back-filled with argon (3 x), and DIPEA (78 µL, 0.45 mmol, 5.0 equiv.) was added *via* syringe. After 3 h, the solution containing the activated carboxylic acid was transferred to the second vial *via* syringe and stirred for 16 h at room temperature. The reaction solution was used directly for flash column chromatography (9 g silica, flow rate 20 mL / min, EtOAc / LP, 10 : 90 to 22 : 78 in 9 min, then 22 : 78 isocratically for 6 min, then to 62 : 38 in 30 min) to give a mixture of the targeted compound **1**, as well as β-γ double bond isomerization compound **1'** (approximate ratio 3 : 1, by NMR, 34.4 mg). Thus, a new reaction vessel was charged with a stirring bar and part of the so obtained material (24.7 mg, 0.052 mmol), evacuated and back-filled with argon. To this was then added *tert*-BuOK (2.9 mg, 0.026 mmol) in dry THF (1.0 mL) *via* syringe and the solution stirred at room temperature for 18 h.

((2*S*,3*R*,4*R*)-4-(3,4-Dimethoxybenzyl)-2-(3,4-dimethoxyphenyl)tetrahydrofuran-3-yl)methyl cyclopent-1-enecarboxylate (**2**)

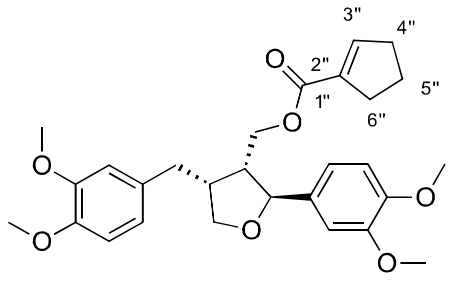

Analogous to 1, using starting material **18** (35.0 mg, 0.090 mmol, 1.00 equiv.) and cyclopent-1-enecarboxylic acid (40.4 mg, 0.360 mmol, 4.0 equiv.), but stirred for 24 in place of 16 h. The reaction solution was used directly for flash column chromatography (9 g silica, flow rate 20 mL / min, EtOAc / LP, 10 : 90 to 22 : 78 in 9 min, then 22 : 78 isocratically for 6 min, then to 62 : 38 in 30 min) to afford the title compound **2**.

**Yield**:	39.4 mg, 91 %
**Appearance**:	colorless oil
***R*$_f$ (silica)**:	0.43 (EtOAc / LP, 1 : 1)
**[α]$_D^{20}$**:	+20.0 (c 3.49, MeOH)
**LC-HRMS (ESI)**:	calculated for M+Na$^+$: 505.2197, found: 505.2200, Δ: 0.59 ppm
**(log *P*)$_{calc}$**:	5.38 ± 0.45

GC-MS (EI, 70 eV, Method E): 37.45 min; 482.2 (M$^+$, 2), 219.1 (28), 207.1 (17), 189.1 (16), 177.1 (16), 165.1 (81), 152.1 (15), 151.1 (100), 107.1 (17), 95.1 (73).

^{1}H NMR (200 MHz, CDCl$_3$): δ 1.95 (quint, ^3J = 7.4 Hz, 2H, H5'''), 2.40 – 2.85 (m, 7H, H3, H4, C4-CH, 2 x H4''', 2 x H6'''), 2.89 (dd, ^2J = 12.4 Hz, ^3J = 4.3 Hz, 1H, C4-CH), 3.76 (dd, ^2J = 8.6 Hz, ^3J = 6.4 Hz, 1H, H5), 3.86 (s, 3H, Ar-OCH$_3$), 3.87 (s, 6H, Ar-OCH$_3$), 3.88 (s, 3H, Ar-OCH$_3$), 4.09 (dd, ^2J = 8.6 Hz, ^3J = 6.3 Hz, 1H, H5), 4.27 (dd, ^2J = 11.2 Hz, ^3J = 7.0 Hz, 1H, C3-CH), 4.43 (dd, ^2J = 11.3 Hz, ^3J = 6.7 Hz, 1H, C3-CH), 4.83 (d, ^3J = 6.3 Hz, 1H, H2), 6.63 – 6.91 (m, 7H, 6 x Ar-H, H3''').

^{13}C NMR (50 MHz, CDCl$_3$): δ 23.2 (t, C5'''), 31.4 (t, C4'''*), 33.3 (t, C4-C), 33.5 (t, C6'''*), 42.7 (d, C4), 49.2 (d, C3), 56.0 (q, 4 x Ar-OCH$_3$), 62.5 (t, C3-C), 72.9 (t, C5), 83.2 (d, C2), 109.0 (d, C2'), 111.1 (d, C5'), 111.4 (d, C5''*), 112.0 (d, C2''*), 118.2 (d, C6'), 120.5 (d, C6''), 132.8 (s, C1''), 135.1 (s, C1'), 136.3 (s, C2'''), 144.5 (d, C3'''), 147.6 (s, C4''), 148.5 (s, C4'), 149.0 (s, C3''), 149.1 (s, C3'), 165.2 (s, C1''').

((2*S*,3*R*,4*R*)-4-(3,4-Dimethoxybenzyl)-2-(3,4-dimethoxyphenyl)tetrahydrofuran-3-yl)methyl cyclohex-1-enecarboxylate (**3**)

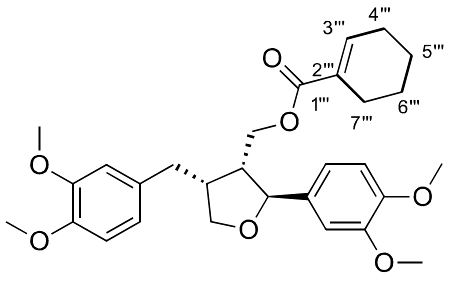

Analogous to **1**, using starting material **18** (35.0 mg, 0.090 mmol, 1.00 equiv.) and cyclohex-1-enecarboxylic acid (45.4 mg, 0.360 mmol, 4.0 equiv.), but stirred for 24 in place of 16 h. The reaction solution was used directly for flash column chromatography (9 g silica, flow rate 20 mL / min, EtOAc / LP, 10 : 90 to 22 : 78 in 9 min, then 22 : 78 isocratically for 6 min, then to 62 : 38 in 30 min) to afford the title compound **3**.

**Yield**:	27.0 mg, 60 %
**Appearance**:	colorless oil
***R*$_f$ (silica)**:	0.47 (EtOAc / LP, 1 : 1)
**$[\alpha]_D^{20}$**:	+20.5 (c 2.64, MeOH)
**LC-HRMS (ESI)**:	calculated for M+Na$^+$: 519.2353, found: 519.2378, Δ: 4.81 ppm
**(log *P*)$_{calc}$**:	5.95 ± 0.45

GC-MS (EI, 70 eV, Method E): 48.95 min; 496.3 (M$^+$, 1), 370.2 (15), 219.1 (32), 207.0 (24), 206.1 (16), 189.1 (17), 177.1 (17), 166.1 (15), 165.1 (89), 152.1 (16), 151.1 (100), 109.1 (39), 107.1 (19).

^{1}H NMR (200 MHz, CDCl$_3$): δ 1.51 – 1.71 (m, 4H, 2 x H5''', 2 x H6'''), 2.10 – 2.27 (m, 4H, 2 x H4''', 2 x H7'''), 2.50 – 2.95 (m, 4H, H3, H4, C4-CH$_2$), 3.76 (dd, ^2J = 8.5 Hz, ^3J = 6.2 Hz, 1H, H5), 3.86 (s, 3H, Ar-OCH$_3$), 3.87 (s, 6H, Ar-OCH$_3$), 3.88 (s, 3H, Ar-OCH$_3$), 4.08 (dd, ^2J = 8.5 Hz, ^3J = 6.2 Hz, 1H, H5), 4.26 (dd, ^2J = 11.3 Hz, ^3J = 7.1 Hz, 1H, C3-CH), 4.42 (dd, ^2J = 11.3 Hz, ^3J = 6.5 Hz, 1H, C3-CH), 4.83 (d, ^3J = 6.3 Hz, 1H, H2), 6.67 – 6.92 (m, 7H, 6 x Ar-H, H3''').

^{13}C NMR (50 MHz, CDCl$_3$): δ 21.5 (d, C6'''*), 22.1 (d, C5'''*), 24.2 (d, C7'''*), 25.9 (d, C4'''*), 33.4 (t, C4-C), 42.8 (d, C4), 49.2 (d, C3), 56.00 (q, Ar-OCH$_3$), 56.02 (q, Ar-OCH$_3$), 56.03 (q, Ar-OCH$_3$), 56.04 (q, Ar-OCH$_3$), 62.6 (t, C3-C), 72.9 (t, C5), 83.3 (d, C2), 109.1 (d, C2'), 111.1 (d, C5'), 111.4 (d, C5''*), 112.0 (d, C2''*), 118.3 (d, C6'), 120.6 (d, C6''), 130.1 (s, C2'''), 132.8 (s, C1''), 135.1 (s, C1'), 140.5 (d, C3'''), 147.6 (s, C4''), 148.5 (s, C4'), 149.1 (s, C3''), 149.1 (s, C3'), 167.5 (s, C1''').

((2*S*,3*R*,4*R*)-4-(3,4-Dimethoxybenzyl)-2-(3,4-dimethoxyphenyl)tetrahydrofuran-3- yl)methyl benzoate **(4)**

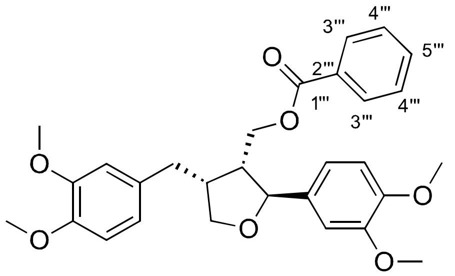

Analogous to <u>**1**</u>, using starting material **18** (35.0 mg, 0.090 mmol, 1.00 equiv.) and benzoic acid (44.0 mg, 0.360 mmol, 4.0 equiv.). The reaction solution was used directly for flash column chromatography (9 g silica, flow rate 20 mL / min, EtOAc / LP, 10 : 90 to 22 : 78 in 9 min, then 22 : 78 isocratically for 6 min, then to 62 : 38 in 30 min) to afford the title compound <u>**4**</u>.

**Yield**:	40.4 mg, 91 %
**Appearance**:	colorless oil
***R*$_f$ (silica)**:	0.54 (EtOAc / LP, 1 : 1)
**$[\alpha]_D^{20}$**:	+20.2 (c 2.04, MeOH)
**LC-HRMS (ESI)**:	calculated for M+Na$^+$: 515.2040, found: 515.2050, Δ: 1.94 ppm
**(log *P*)$_{calc}$**:	5.70 ± 0.44

GC-MS (EI, 70 eV, Method E): 46.13 min; 492.2 (M$^+$, 3), 219.1 (24), 207.0 (24), 189.1 (16), 177.1 (15), 165.1 (72), 151.1 (94), 107.1 (18), 106.1 (15), 105.0 (100).

^{1}H NMR (200 MHz, $CDCl_3$): δ 2.55 – 3.02 (m, 4H, H3, H4, C4-CH_2), 3.76 – 3.83 (m, 1H, H5), 3.84 (s, 3H, Ar-OCH_3), 3.85 (s, 6H, Ar-OCH_3), 3.86 (s, 3H, Ar-OCH_3), 4.13 (dd, ^2J = 8.6 Hz, ^3J = 6.1 Hz, 1H, H5), 4.46 (dd, ^2J = 11.2 Hz, ^3J = 7.1 Hz, 1H, C3-CH), 4.63 (dd, ^2J = 11.3 Hz, ^3J = 6.3 Hz, 1H, C3-CH), 4.91 (d, ^3J = 6.2 Hz, 1H, H2), 6.66 – 6.95 (m, 6H, 3 x Ar'-H, 3 x Ar''-H), 7.42 (t, ^3J = 7.4 Hz, 2H, H4'''), 7.50 – 7.62 (m, 1H, H5'''), 7.89 – 7.97 (m, 2H, H3''').

^{13}C NMR (50 MHz, $CDCl_3$): δ 33.5 (t, C4-<u>C</u>), 42.8 (d, C4), 49.3 (d, C3), 55.9 (q, Ar-OCH_3), 55.99 (q, Ar-OCH_3), 56.02 (q, Ar-OCH_3), 56.1 (q, Ar-OCH_3), 63.4 (t, C3-<u>C</u>), 73.0 (t, C5), 83.5 (d, C2), 109.1 (d, C2'), 111.2 (d, C5'), 111.4 (d, C5''*), 112.0 (d, C2''*), 118.3 (d, C6'), 120.6 (d, C6''), 128.5 (d, C4'''), 129.7 (d, C3'''), 130.0 (s, C2'''), 132.6 (s, C1''), 133.3 (d, C5'''), 135.0 (s, C1'), 147.7 (s, C4''), 148.6 (s, C4'), 149.1 (s, C3''), 149.2 (s, C3'), 166.5 (s, C1''').

((2*S*,3*R*,4*R*)-4-(3,4-Dimethoxybenzyl)-2-(3,4-dimethoxyphenyl)tetrahydrofuran-3-yl)methyl cyclohexanecarboxylate (**5**)

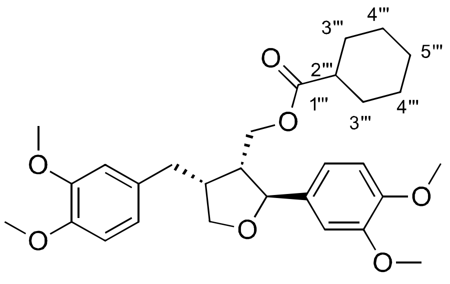

Analogous to **1**, using starting material **18** (35.0 mg, 0.090 mmol, 1.00 equiv.), cyclohexanecarboxylic acid (26.5 mg, 0.207 mmol, 2.3 equiv.), EDCI.HCl (34.5 mg, 0.180 mmol, 2.0 equiv.), 4-DMAP (1.1 mg, 9.0 µmol, 0.1 equiv.) and DIPEA (39 µL, 0.23 mmol, 2.5 equiv.). The reaction solution was used directly for flash column chromatography (9 g silica, flow rate 20 mL / min, EtOAc / LP, 10 : 90 to 22 : 78 in 9 min, then 22 : 78 isocratically for 6 min, then to 62 : 38 in 30 min) to afford the title compound **5**.

**Yield**:	33.3 mg, 74 %
**Appearance**:	colorless oil
***R*f (silica)**:	0.62 (EtOAc / LP, 1 : 1)
**$[\alpha]_D^{20}$**:	+16.8 (c 1.62, MeOH)
**LC-HRMS (ESI)**:	calculated for $M+Na^+$: 521.2510, found: 521.2516, Δ: 1.15 ppm
**(log *P*)calc**:	5.75 ± 0.44

GC-MS (EI, 70 eV, Method D): 40.73 min; 498.2 (M^+, 3), 219.1 (26), 165.1 (53), 151.1 (100).

^{1}H NMR (200 MHz, $CDCl_3$): δ 1.12 – 1.96 (m, 10H, 4 x H3''', 4 x H4''', 2 x H5'''), 2.18 – 2.35 (m, 1H, H2'''), 2.46 – 2.83 (m, 3H, H3, H4, C4-CH), 2.86 (dd, ^2J = 12.4 Hz, ^3J = 4.3 Hz, 1H, C4-CH), 3.76 (dd, ^2J = 8.6 Hz, ^3J = 6.2 Hz, 1H, H5), 3.87 (s, 3H, Ar-OCH_3), 3.87 (s, 6H, Ar-OCH_3), 3.89 (s, 3H, Ar-OCH_3), 4.07 (dd, ^2J = 8.6 Hz, ^3J = 6.3 Hz, 1H, H5), 4.17 (dd, ^2J = 11.3 Hz, ^3J = 6.9 Hz, 1H, C3-CH), 4.37 (dd, ^2J = 11.3 Hz, ^3J = 6.9 Hz, 1H, C3-CH), 4.80 (d, ^3J = 6.4 Hz, 1H, H2), 6.66 – 6.91 (m, 6H, Ar-H).

^{13}C NMR (50 MHz, $CDCl_3$): δ 25.5 (t, 2 x C4'''), 25.8 (t, C5'''), 29.1 (t, 2 x C3'''), 29.1 (t, C3'''), 33.3 (t, C4-C), 42.7 (d, C4), 43.3 (d, C2'''), 49.3 (d, C3), 56.0 (q, 4 x Ar-OCH_3), 62.4 (t, C3-C), 72.9 (t, C5), 83.0 (d, C2), 109.0 (d, C2'), 111.1 (d, C5'), 111.4 (d, C5''*), 112.0 (d, C2''*), 118.2 (d, C6'), 120.6 (d, C6''), 132.7 (s, C1''), 135.1 (s, C1'), 147.6 (s, C4''), 148.6 (s, C4'), 149.1 (s, C3''), 149.2 (s, C3'), 176.0 (s, C1''').

((2*S*,3*R*,4*R*)-4-(3,4-Dimethoxybenzyl)-2-(3,4-dimethoxyphenyl)tetrahydrofuran-3- yl)methyl cyclopentanecarboxylate (**6**)

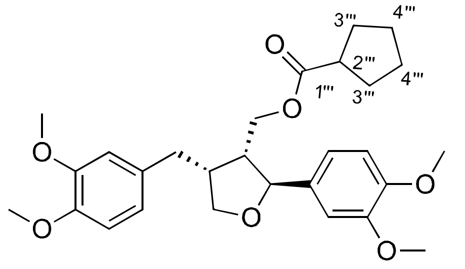

Analogous to **1** using starting material **18** (35.0 mg, 0.090 mmol, 1.00 equiv.), cyclopentanecarboxylic acid (23.6 mg, 0.207 mmol, 2.3 equiv.), EDCI.HCl (34.5 mg, 0.180 mmol, 2.0 equiv.), 4-DMAP (1.1 mg, 9.0 µmol, 0.1 equiv.) and DIPEA (39 µL, 0.23 mmol, 2.5 equiv.). The reaction solution was used directly for flash column chromatography (9 g silica, flow rate 20 mL / min, EtOAc / LP, 10 : 90 to 22 : 78 in 9 min, then 22 : 78 isocratically for 6 min, then to 62 : 38 in 30 min) to afford the title compound **6**.

Yield: 38.7 mg, 89 %

Appearance: colorless oil

***R*f (silica)**: 0.59 (EtOAc / LP, 1 : 1)

[α]D20: +17.4 (c 2.47, MeOH)

LC-HRMS (ESI): calculated for M+Na+: 507.2353, found: 507.2358, Δ: 0.99 ppm

(log *P*)calc: 5.18 ± 0.44

GC-MS (EI, 70 eV, Method F): 33.28 min; 484.2 (M+, 3), 219.1 (24), 165.1 (57), 151.0 (100).

^{1}H NMR (200 MHz, $CDCl_3$): δ 1.48 – 1.97 (m, 8H, 4 x H3''', 4 x H4'''), 2.47 – 2.93 (m, 5H, H3, H4, C4-CH_2, H2'''), 3.76 (dd, ^2J = 8.5 Hz, ^3J = 6.3 Hz, 1H, H5), 3.87 (s, 3H, Ar-OCH_3), 3.87 (s, 6H, Ar-OCH_3), 3.89 (s, 3H, Ar-OCH_3), 4.07 (dd, ^2J = 8.6 Hz, ^3J = 6.2 Hz, 1H, H5), 4.18 (dd, ^2J = 11.3 Hz, ^3J = 7.0 Hz, 1H, C3-CH), 4.37 (dd, ^2J = 11.2 Hz, ^3J = 6.9 Hz, 1H, C3-CH), 4.80 (d, ^3J = 6.3 Hz, 1H, H2), 6.66 – 6.91 (m, 6H, Ar-H).

^{13}C NMR (50 MHz, $CDCl_3$): δ 25.9 (t, 2 x C4'''), 30.1 (t, 2 x C3'''), 33.3 (t, C4-C), 42.7 (d, C4), 44.0 (d, C2'''), 49.3 (d, C3), 56.0 (q, 4 x Ar-OCH_3), 62.6 (t, C3-C), 72.8 (t, C5), 83.0 (d, C2), 109.0 (d, C2'), 111.1 (d, C5'), 111.4 (d, C5''*), 112.0 (d, C2''*), 118.2 (d, C6'), 120.6 (d, C6''), 132.7 (s, C1''), 135.1 (s, C1'), 147.6 (s, C4''), 148.6 (s, C4'), 149.0 (s, C3''), 149.2 (s, C3'), 176.7(s, C1''').

((2*S*,3*R*,4*R*)-4-(3,4-Dimethoxybenzyl)-2-(3,4-dimethoxyphenyl)tetrahydrofuran-3- yl)methyl cyclobutanecarboxylate (**7**)

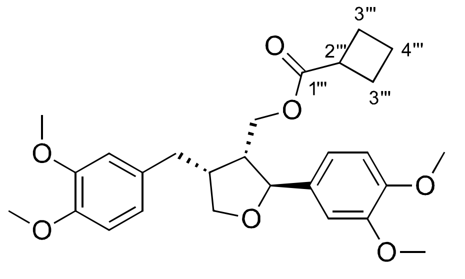

Analogous to **1**, using starting material **18** (21.5 mg, 0.055 mmol, 1.00 equiv.), cyclobutanecarboxylic acid (12.7 mg, 0.127 mmol, 2.3 equiv.), EDCI.HCl (21.2 mg, 0.111 mmol, 2.0 equiv.), 4-DMAP (0.7 mg, 5.5 µmol, 0.1 equiv.) and DIPEA (24 µL, 0.14 mmol, 2.5 equiv.), and stirring the reaction for 24 in place of 16 h. The reaction solution was used directly for flash column chromatography (9 g silica, flow rate 20 mL / min, EtOAc / LP, 10 : 90 to 15 : 85 in 10 min, then to 25 : 75 in 5 min, then to 50 : 50 in 7 min) to afford the title compound **7**.

Yield: 21.5 mg, 83 %

Appearance: colorless oil

R_f (silica): 0.37 (EtOAc / heptane, 1 : 1)

$[\alpha]_D^{23}$: +22.8 (c 1.27, MeOH)

LC-HRMS (APCI): calculated for M+Na$^+$: 493.2197, found: 493.2214, Δ: 3.45 ppm

$(\log P)_{calc}$: 4.62 ± 0.44

GC-MS (EI, 70 eV, Method D): 22.60 min; 470.1 (M$^+$, 4), 207.0 (52), 177.1 (16), 166.1 (17), 165.1 (50), 151.0 (100), 107.1 (15).

^{1}H NMR (200 MHz, CDCl$_3$): δ 1.79 – 2.06 (m, 2H, H4'''), 2.07 – 2.37 (m, 4H, H3'''), 2.47 – 2.93 (m, 4H, H3, H4, C4-CH$_2$), 3.11 (quint, ^3J = 8.6 Hz, 1H, H2'''), 3.75 (dd, ^2J = 8.5 Hz, ^3J = 6.3 Hz, 1H, H5), 3.86 (s, 3H, Ar-OCH$_3$), 3.87 (s, 6H, Ar-OCH$_3$), 3.89 (s, 3H, Ar-OCH$_3$), 4.07 (dd, ^2J = 8.5 Hz, ^3J = 6.3 Hz, 1H, H5), 4.19 (dd, ^2J = 11.2 Hz, ^3J = 7.1 Hz, 1H, C3-CH), 4.38 (dd, ^2J = 11.2 Hz, ^3J = 6.9 Hz, 1H, C3-CH), 4.80 (d, ^3J = 6.4 Hz, 1H, H2), 6.66 – 6.90 (m, 6H, Ar-H).

^{13}C NMR (50 MHz, CDCl$_3$): δ 18.6 (t, C4'''), 25.4 (t, 2 x C3'''), 25.4 (t, C3'''), 33.3 (t, C4-C), 38.2 (d, C2'''), 42.7 (d, C4), 49.2 (d, C3), 56.0 (q, 4 x Ar-OCH$_3$), 62.6 (t, C3-C), 72.9 (t, C5), 83.1 (d, C2), 109.0 (d, C2'), 111.1 (d, C5'), 111.5 (d, C5''*), 112.0 (d, C2''*), 118.2 (d, C6'), 120.6 (d, C6''), 132.7 (s, C1''), 135.1 (s, C1'), 147.6 (s, C4''), 148.6 (s, C4'), 149.1 (s, C3''), 149.2 (s, C3'), 175.4 (s, C1''').

((2*S*,3*R*,4*R*)-4-(3,4-Dimethoxybenzyl)-2-(3,4-dimethoxyphenyl)tetrahydrofuran-3-yl)methylbutyrate (**8**)

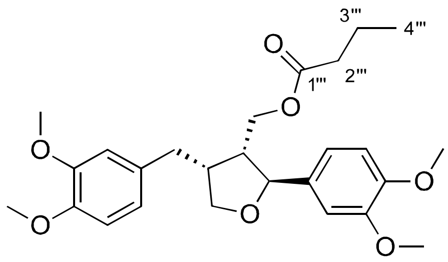

Analogous to **1**, using starting material **18** (35.0 mg, 0.090 mmol, 1.00 equiv.), butyric acid (18.2 mg, 0.207 mmol, 2.3 equiv.), EDCI.HCl (34.5 mg, 0.180 mmol, 2.0 equiv.), 4-DMAP (1.1 mg, 9.0 µmol, 0.1 equiv.) and DIPEA (39 µL, 0.23 mmol, 2.5 equiv.). The reaction solution was used directly for flash column chromatography (9 g silica, flow rate 20 mL / min, EtOAc / LP, 10 : 90 to 22 : 78 in 9 min, then 22 : 78 isocratically for 6 min, then to 62 : 38 in 30 min) to afford the title compound **8**.

**Yield**:	35.1 mg, 85 %
**Appearance**:	colorless oil
***R*$_f$ (silica)**:	0.44 (EtOAc / LP, 1 : 1)
**$[\alpha]_D^{20}$**:	+20.3 (c 1.77, MeOH)
**LC-HRMS (ESI)**:	calculated for $M+Na^+$: 481.2197, found: 481.2207, Δ: 2.08 ppm
**(log *P*)$_{calc}$**:	4.75 ± 0.44

GC-MS (EI, 70 eV, Method D): 20.50 min; 458.2 (M^+, 6), 219.1 (21), 165.1 (52), 152.1 (15), 151.1 (100).

^{1}H NMR (200 MHz, $CDCl_3$): δ 0.94 (t, ^3J = 7.4 Hz, 3H, H4'''), 1.64 (sext, ^3J = 7.4 Hz, 2H, H3'''), 2.26 (t, ^3J = 7.4 Hz, 2H, H2'''), 2.47 – 2.93 (m, 4H, H3, H4, C4-CH_2), 3.75 (dd, ^2J = 8.6 Hz, ^3J = 6.2 Hz, 1H, H5), 3.86 (s, 3H, Ar-OCH_3), 3.87 (s, 6H, Ar-OCH_3), 3.89 (s, 3H, Ar-OCH_3), 4.07 (dd, ^2J = 8.6 Hz, ^3J = 6.3 Hz, 1H, H5), 4.19 (dd, ^2J = 11.2 Hz, ^3J = 7.1 Hz, 1H, C3-CH), 4.38 (dd, ^2J = 11.2 Hz, ^3J = 6.9 Hz, 1H, C3-CH), 4.79 (d, ^3J = 6.4 Hz, 1H, H2), 6.64 – 6.92 (m, 6H, Ar-H).

^{13}C NMR (50 MHz, $CDCl_3$): δ 13.8 (q, C4'''), 18.5 (t, C3'''), 33.3 (t, C4-C), 36.3 (t, C2'''), 42.6 (d, C4), 49.1 (d, C3), 56.0 (q, 4 x Ar-OCH_3), 62.5 (t, C3-C), 72.8 (t, C5), 83.0 (d, C2), 109.0 (d, C2'), 111.1 (d, C5'), 111.4 (d, C5''*), 112.0 (d, C2''*), 118.2 (d, C6'), 120.5 (d, C6''), 132.7 (s, C1''), 135.0 (s, C1'), 147.6 (s, C4''), 148.6 (s, C4'), 149.0 (s, C3''), 149.1 (s, C3'), 173.6 (s, C1''').

((2*S*,3*R*,4*R*)-4-(3,4-Dimethoxybenzyl)-2-(3,4-dimethoxyphenyl)tetrahydrofuran-3-yl)methyl pivalate (**9**)

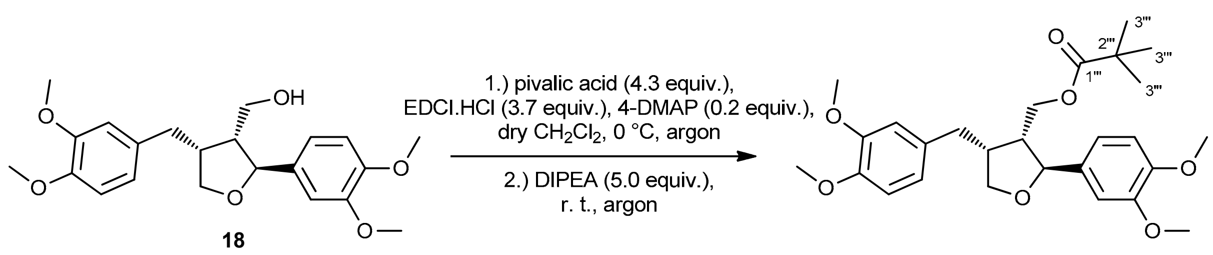

A reaction vessel was charged with a stirring bar, pivalic acid (21.1 mg, 0.207 mmol, 2.3 equiv.) and 4-DMAP (1.1 mg, 9.0 µmol, 0.1 equiv.), and then evacuated and back-filled with argon using standard Schlenk technique. Dry CH_2Cl_2 (1.0 mL) was then added *via* syringe and the solution was cooled to 0 °C in an ice bath. The vessel was briefly opened, EDCI.HCl (34.5 mg, 0.180 mmol, 2.0 equiv.) added in one go and the mixture was stirred for 3 h at 0 °C. Meanwhile, a second vessel was charged with a stirring bar and **18** (35.0 mg, 0.090 mmol, 1.00 equiv.), evacuated and back-filled with argon (3 x), and DIPEA (39 µL, 0.23 mmol, 2.5 equiv.) was added *via* syringe. After 3 h, the solution containing the activated carboxylic acid was transferred to the second vial *via* syringe and stirred for 70 h at room temperature. To complete the reaction, more of the activated carboxylic acid was prepared in a separate vessel in the same way as above (using pivalic acid (18.3 mg, 0.180 mmol, 2.0 equiv.), 4-DMAP (1.1 mg, 9.0 µmol, 0.1 equiv.) and EDCI.HCl (30.0 mg, 0.157 mmol, 1.7 equiv.)) and then, after 3 h at 0 °C, added to the reaction vial, followed by more DIPEA (39 µL, 0.23 mmol, 2.5 equiv.) *via* syringe, and the mixture was stirred for another 96 h at room temperature.The reaction solution was used directly for flash column chromatography (9 g silica, flow rate 20 mL / min, EtOAc / LP, 10 : 90 to 22 : 78 in 9 min, then 22 : 78 isocratically for 6 min, then to 38 : 62 in 12 min, then to 100 : 0 in 10 min) to afford the title compound **9**.

Yield: 32.2 mg, 76 %

Appearance: colorless oil

***R_f* (silica)**: 0.49 (EtOAc / LP, 1 : 1)

$[\alpha]_D^{23}$: +22.5 (c 2.72, MeOH)

LC-HRMS (ESI): calculated for $M+Na^+$: 495.2353, found: 495.2351, Δ: -0.40 ppm

(log *P*)$_{calc}$: 4.91 ± 0.45

GC-MS (EI, 70 eV, Method D): 19.53 min; 472.2 (M^+, 5), 219.1 (25), 165.1 (54), 152.1 (15), 151.1 (100).

^{1}H NMR (200 MHz, $CDCl_3$): δ 1.21 (s, 9H, H3'''), 2.45 – 2.81 (m, 3H, H3, H4, C4-CH), 2.87 (dd, ^2J = 12.4 Hz, ^3J = 4.1 Hz, 1H, C4-CH), 3.77 (dd, ^2J = 8.6 Hz, ^3J = 6.2 Hz, 1H, H5), 3.88 (s, 9H, Ar-OCH_3), 3.89 (s, 3H, Ar-OCH_3), 4.07 (dd, ^2J = 8.6 Hz, ^3J = 6.3 Hz, 1H, H5), 4.17 (dd, ^2J = 11.3 Hz, ^3J = 6.8 Hz, 1H, C3-CH), 4.36 (dd, ^2J = 11.3 Hz, ^3J = 6.9 Hz, 1H, C3-CH), 4.82 (d, ^3J = 6.3 Hz, 1H, H2), 6.66 – 6.90 (m, 6H, Ar-H).

^{13}C NMR (50 MHz, $CDCl_3$): δ 27.3 (q, 3 x C3'''), 33.3 (t, C4-C), 38.9 (s, C2'''), 42.8 (d, C4), 49.4 (d, C3), 55.98 (q, Ar-OCH_3), 56.00 (q, Ar-OCH_3), 56.03 (q, Ar-OCH_3), 56.1 (q, Ar-OCH_3), 62.7 (t, C3-C), 72.8 (t, C5), 82.9 (d, C2), 109.0 (d, C2'), 111.2 (d, C5'), 111.4 (d, C5''*), 112.0 (d, C2''*), 118.1 (d, C6'), 120.6 (d, C6''), 132.7 (s, C1''), 135.1 (s, C1'), 147.6 (s, C4''), 148.6 (s, C4'), 149.1 (s, C3''), 149.2 (s, C3'), 178.5 (s, C1''').

((2*S*,3*R*,4*R*)-4-(3,4-Dimethoxybenzyl)-2-(3,4-dimethoxyphenyl)tetrahydrofuran-3-yl)methyl 3,3-dimethylbutanoate (**10**)

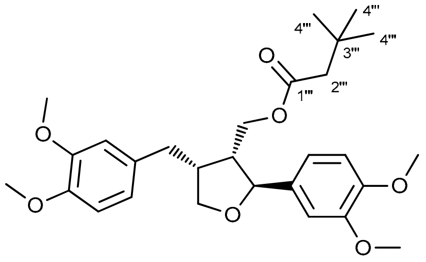

Analogous to **1**, using starting material **18** (30.0 mg, 0.077 mmol, 1.00 equiv.) and 3,3-dimethylbutanoic acid (20.6 mg, 0.178 mmol, 2.3 equiv.), EDCI.HCl (29.6 mg, 0.154 mmol, 2.0 equiv.), 4-DMAP (0.9 mg, 7.7 µmol, 0.1 equiv.) and DIPEA (34 µL, 0.19 mmol, 2.5 equiv.).The reaction solution was used directly for flash column chromatography (9 g silica, flow rate 20 mL / min, EtOAc / LP, 10 : 90 to 30 : 70 in 30 min) to afford the title compound **10**.

**Yield**:	19.5 mg, 51 %
**Appearance**:	colorless oil
***R*$_f$ (silica)**:	0.43 (EtOAc / heptane, 1 : 1)
**$[\alpha]_D^{23}$**:	+24.7 (c 1.01, MeOH)
**LC-HRMS (APCI)**:	calculated for $M+Na^+$: 509.2510, found: 509.2512, Δ: 0.39 ppm
**(log *P*)$_{calc}$**:	5.45 ± 0.45

GC-MS (EI, 70 eV, Method D): 18.75 min; 486.1 (M^+, 4), 219.0 (17), 166.0 (17), 165.0 (45), 152.1 (15), 151.0 (100).

^{1}H NMR (200 MHz, CDCl$_3$): δ 1.03 (s, 9H, H4'''), 2.20 (s, 2H, H2'''), 2.47 – 2.63 (m, 2H, H3, C4-CH), 2.64 – 2.82 (m, 1H, H4), 2.88 (dd, ^2J = 12.5 Hz, ^3J = 4.2 Hz, 1H, C4-CH), 3.76 (dd, ^2J = 8.6 Hz, ^3J = 6.1 Hz, 1H, H5), 3.86 (s, 3H, Ar-OCH$_3$), 3.87 (s, 3H, Ar-OCH$_3$), 3.87 (s, 3H, Ar-OCH$_3$), 3.89 (s, 3H, Ar-OCH$_3$), 4.06 (dd, ^2J = 8.6 Hz, ^3J = 6.2 Hz, 1H, H5), 4.16 (dd, ^2J = 11.3 Hz, ^3J = 6.9 Hz, 1H, C3-CH), 4.36 (dd, ^2J = 11.3 Hz, ^3J = 7.2 Hz, 1H, C3-CH), 4.80 (d, ^3J = 6.4 Hz, 1H, H2), 6.66 – 6.90 (m, 6H, Ar-H).

^{13}C NMR (50 MHz, CDCl$_3$): δ 29.8 (q, 3 x C4'''), 30.9 (s, C3'''), 33.2 (t, C4-C), 42.6 (d, C4), 48.1 (t, C2'''), 49.3 (d, C3), 56.0 (q, 4 x Ar-OCH$_3$), 62.3 (t, C3-C), 72.8 (t, C5), 82.9 (d, C2), 109.0 (d, C2'), 111.2 (d, C5'), 111.5 (d, C5''*), 112.0 (d, C2''*), 118.2 (d, C6'), 120.6 (d, C6''), 132.7 (s, C1''), 135.1 (s, C1'), 147.7 (s, C4''), 148.6 (s, C4'), 149.1 (s, C3''), 149.2 (s, C3'), 172.4 (s, C1''').

((2*S*,3*R*,4*R*)-4-(3,4-Dimethoxybenzyl)-2-(3,4-dimethoxyphenyl)tetrahydrofuran-3-yl)methyl2-ethylbutanoate (**11**)

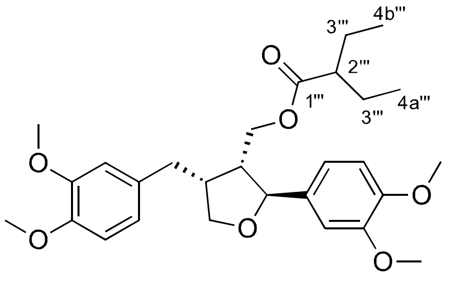

Analogous to **1**, using starting material **18** (20.3 mg, 0.052 mmol, 1.00 equiv.) and 2-ethylbutanoic acid (14.0 mg, 0.120 mmol, 2.3 equiv.), EDCI.HCl (20.0 mg, 0.105 mmol, 2.0 equiv.), 4-DMAP (0.6 mg, 5.2 µmol, 0.1 equiv.) and DIPEA (23 µL, 0.13 mmol, 2.5 equiv.). The reaction solution was used directly for flash column chromatography (9 g silica, flow rate 20 mL / min, EtOAc / LP, 10 : 90 to 30 : 70 in 30 min) to afford the title compound **11**.

Yield: 22.2 mg, 87 %

Appearance: colorless oil

***R*$_f$ (silica)**: 0.44 (EtOAc / heptane, 1 : 1)

$[\alpha]_D^{23}$: +23.3 (c 0.88, MeOH)

LC-HRMS (APCI): calculated for M+Na$^+$: 509.2510, found: 509.2533, Δ: 4.52 ppm

(log *P*)$_{calc}$: 5.63 ± 0.45

GC-MS (EI, 70 eV, Method E): 23.47 min; 486.2 (M$^+$, 7), 219.1 (32), 189.1 (15), 165.1 (56), 151.0 (100).

^{1}H NMR (200 MHz, CDCl$_3$): δ 0.90 (t, ^3J = 7.4 Hz, 6H, 3 x H4a''', 3 x H4b'''), 1.42 – 1.75 (m, 4H, H3'''), 2.15 – 2.30 (m, 1H, H2'''), 2.47 – 2.82 (m, 3H, H3, H4, C4-CH), 2.88 (dd, ^2J = 12.6 Hz, ^3J = 4.0 Hz, 1H, C4-CH), 3.76 (dd, ^2J = 8.6 Hz, ^3J = 6.1 Hz, 1H, H5), 3.87 (s, 6H, Ar-OCH$_3$), 3.87 (s, 3H, Ar-OCH$_3$), 3.89 (s, 3H, Ar-OCH$_3$), 4.06 (dd, ^2J = 8.6 Hz, ^3J = 6.2 Hz, 1H, H5), 4.18 (dd, ^2J = 11.3 Hz, ^3J = 6.8 Hz, 1H, C3-CH), 4.41 (dd, ^2J = 11.3 Hz, ^3J = 7.1 Hz, 1H, C3-CH), 4.81 (d, ^3J = 6.4 Hz, 1H, H2), 6.64 – 6.91 (m, 6H, Ar-H).

^{13}C NMR (50 MHz, CDCl$_3$): δ 12.0 (q, C4a'''*), 12.1 (q, C4b'''*), 25.1 (t, 2 x C3'''), 33.2 (t, C4-C), 42.7 (d, C4), 49.2 (d, C3*), 49.4 (d, C2'''*), 55.99 (q, Ar-OCH$_3$), 56.01 (q, Ar-OCH$_3$), 56.04 (q, Ar-OCH$_3$), 56.1 (q, Ar-OCH$_3$), 62.4 (t, C3-C), 72.8 (t, C5), 83.0 (d, C2), 109.0 (d, C2'), 111.1 (d, C5'), 111.5 (d, C5''*), 112.0 (d, C2''*), 118.2 (d, C6'), 120.6 (d, C6''), 132.7 (s, C1''), 135.1 (s, C1'), 147.6 (s, C4''), 148.6 (s, C4'), 149.1 (s, C3''), 149.2 (s, C3'), 176.2 (s, C1''').

((2*S*,3*R*,4*R*)-4-(3,4-Dimethoxybenzyl)-2-(3,4-dimethoxyphenyl)tetrahydrofuran-3-yl)methyl cycloheptanecarboxylate (**12**)

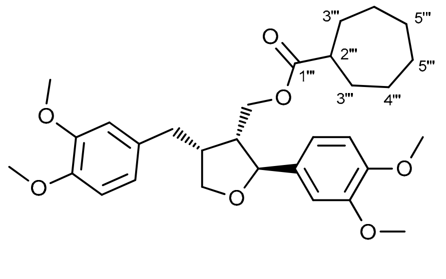

Analogous to **1**, using starting material **18** (35.0 mg, 0.090 mmol, 1.00 equiv.), cycloheptanecarboxylic acid (29.4 mg, 0.207 mmol, 2.3 equiv.), EDCI.HCl (34.5 mg, 0.180 mmol, 2.0 equiv.), 4-DMAP (1.1 mg, 9.0 µmol, 0.1 equiv.) and DIPEA (39 µL, 0.23 mmol, 2.5 equiv.). The reaction solution was used directly for flash column chromatography (9 g silica, flow rate 20 mL / min, EtOAc / LP, 10 : 90 to 15 : 85 in 10 min, then to 25 : 75 in 7 min, then to 50 : 50 in 12 min) to afford the title compound **12**.

Yield: 38.8 mg, 84 %

Appearance: colorless oil

***R*$_f$ (silica)**: 0.53 (EtOAc / LP, 1 : 1)

$[\alpha]_D^{25}$: +20.0 (c 3.74, MeOH)

LC-HRMS (ESI): calculated for $M+H^+$: 513.2847, found: 513.2842, Δ: -0.87 ppm

(log *P*)$_{calc}$: 6.31 ± 0.44

^{1}H NMR (200 MHz, $CDCl_3$): δ 1.36 – 1.80 (m, 10H, 2 x H3''', 4 x H4''', 4 x H5'''), 1.80 – 1.99 (m, 2H, 2 x H3'''), 2.36 – 2.82 (m, 4H, H3, H4, C4-CH, H2'''), 2.86 (dd, ^2J = 12.4 Hz, ^3J = 4.1 Hz, 1H, C4-CH), 3.76 (dd, ^2J = 8.6 Hz, ^3J = 6.1 Hz, 1H, H5), 3.87 (s, 3H, Ar-OCH_3), 3.87 (s, 6H, Ar-OCH_3), 3.89 (s, 3H, Ar-OCH_3), 4.07 (dd, ^2J = 8.6 Hz, ^3J = 6.3 Hz, 1H, H5), 4.16 (dd, ^2J = 11.3 Hz, ^3J = 7.0 Hz, 1H, C3-CH), 4.37 (dd, ^2J = 11.2 Hz, ^3J = 6.9 Hz, 1H, C3-CH), 4.80 (d, ^3J = 6.4 Hz, 1H, H2), 6.66 – 6.90 (m, 6H, Ar-H).

^{13}C NMR (50 MHz, $CDCl_3$): δ 26.4 (t, 2 x C4'''), 28.4 (t, 2 x C5'''), 30.9 (t, 2 x C3'''), 33.3 (t, C4-C), 42.7 (d, C4), 45.2 (d, C2'''), 49.3 (d, C3), 56.0 (q, 4 x Ar-OCH_3), 62.5 (t, C3-C), 72.8 (t, C5), 83.1 (d, C2), 109.0 (d, C2'), 111.1 (d, C5'), 111.4 (d, C5''*), 112.0 (d, C2''*), 118.2 (d, C6'), 120.6 (d, C6''), 132.7 (s, C1''), 135.1 (s, C1'), 147.6 (s, C4''), 148.6 (s, C4'), 149.1 (s, C3''), 149.2 (s, C3'), 176.9 (s, C1''').

((2S,3R,4R)-4-(3,4-Dimethoxybenzyl)-2-(3,4,5-trimethoxyphenyl)tetrahydrofuran-3-yl)methyl butyrate **(13)**

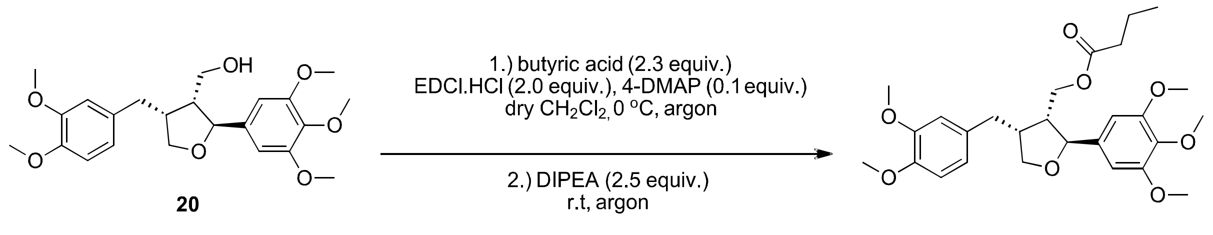

Analogous to **1**, using starting material **20** (35.0 mg, 0.090 mmol, 1.00 equiv.), butyric acid (29.4 mg, 0.207 mmol, 2.3 equiv.), EDCI.HCl (34.5 mg, 0.180 mmol, 2.0 equiv.), 4-DMAP (1.1 mg, 9.0 µmol, 0.1 equiv.) and DIPEA (39 µL, 0.23 mmol, 2.5 equiv.). The reaction solution was used directly for flash column chromatography (9 g silica, flow rate 20 mL / min, , first 10-22 % EtOAc in 10 min, then 22 % EtOAc for 6 min, then 22 - 65 % in 30 min) to afford the title compound **13**.

Yield: 25.1 mg (72 %)

Appearance: slightly yellow viscous oil

***R*$_f$ (silica)**: 0.66(EtOAc / LP, 1 : 1)

$[\alpha]_D^{20}$: +19.2 (c 1.948, MeOH)

HRMS (ESI$^+$): exact mass calculated for $C_{27}H_{36}O_8$: 511.2302. Found: 511.2282. $[M+Na]^+$, Δ = 3.91 ppm

GC-MS (EI, 70 eV, Method B): 515 (M^+, 12), 249 (26), 203 (12), 196 (10), 195 (61), 190 (11), 189 (15), 181 (23), 178 (10), 177 (14), 159 (10), 152 (14), 151 (100), 107 (18)

^{1}H-NMR (200 MHz, CDCl$_3$): δ 0.94 (t, J=7.43, 3H, CH_3), 1.64 (tq, J=7.43 & 7.44, 2H, <u>CH_2</u>CH_3), 2.26 (t, J=7.44, 2H, (CO)<u>CH_2</u>), 2.46-2-92 (m, 4H, H3 & H4 & CH_2), 3.74 (dd, J=8.61 & 6.45 Hz, 1H, H5), 3.82 (s, 3H, OCH_3), 3.85 (s, 9H, 3xOCH_3), 3.88 (s, 3H, OCH_3), 4.06 (dd, J=8.61 & 6.26 Hz, 1H, H5), 4.20 (dd, J=11.15 & 7.24 Hz, 1H, CH_2O), 4.38 (dd, J=11.15 & 6.75 Hz, CH_2O), 4.77 (d, J=5.87 Hz, 1H, H2), 6.53 (s, 2H, H2' & H6'), 6.64-6.83 (m, 3H, H2'' & H5'' & H6'')

^{13}C-NMR (50 MHz, CDCl$_3$): δ 13.7 (q, CH_3), 18.4 (t, <u>CH_2</u>CH_3), 33.1 (t, CH_2), 36.2 (t, (CO)<u>CH_2</u>), 42.4 (d, C4), 49.0 (d, C3), 55.9 (q, 2xOCH_3), 56.1 (q, 2xOCH_3), 60.8 (q, OCH_3), 62.5 (t, CH_2O), 72.8 (t, C5), 83.2 (d, C2), 102.6 (d, C2' & C6'), 111.3 (d, C2''), 111.8 (d, C5''), 120.4 (d, C6''), 132.5 (s, C1''), 138.2 (s, C_q), 147.5 (s, C4''), 148.9 (s, C3''), 153.3 (s, C3' & C5'), 173.5 (s, C=O). One C_q not visible.

((2*S*,3*R*,4*R*)-4-(3,4-Dimethoxybenzyl)-2-(4-fluorophenyl)tetrahydrofuran-3-yl)methyl 3,3-dimethylbutanoate (**<u>14</u>**)

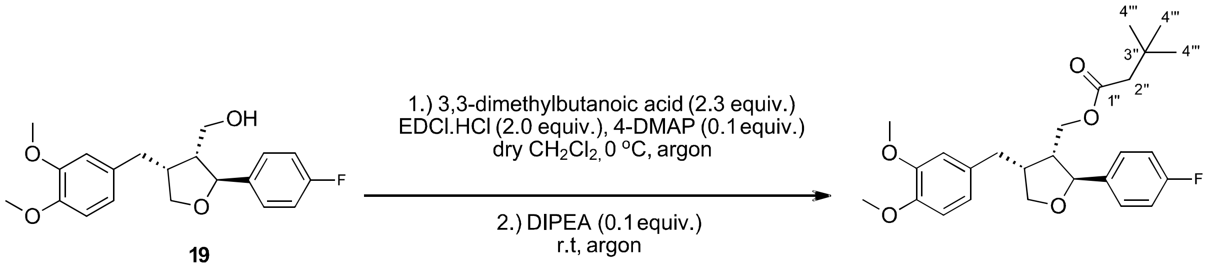

Analogous to **<u>1</u>**, using starting material **<u>19</u>** (22.0 mg, 0.064 mmol, 1.00 equiv.), 3,3-dimethylbutanoic acid (16.9 mg, 0.146 mmol, 2.3 equiv.), EDCI.HCl (24.3 mg, 0.127 mmol, 2.0 equiv.), 4-DMAP (0.8 mg, 6.4 µmol, 0.1 equiv.) and DIPEA (28 µL, 0.16 mmol, 2.5 equiv.), and stirring for 13.5 in place of 16 h. The reaction solution was used directly for flash column chromatography (9 g silica, flow rate 20 mL / min, EtOAc / LP, 9 : 91 to 25 : 75 in 60 min) to afford the title compound **<u>14</u>**.

Yield: 25.8 mg, 92 %

Appearance: colorless oil

***R*$_f$ (silica)**: 0.66 (EtOAc / heptane, 1 : 1)

$[\alpha]_D^{23}$: +15.7 (c 2.07, MeOH)

LC-HRMS (APCI): calculated for $M+H^+$: 445.2385, found: 445.2398, Δ: 2.92 ppm

(log *P*)$_{calc}$: 5.76 ± 0.50

GC-MS (EI, 70 eV, Method D): 11.19 min; 444.1 (M^+, 7), 194.0 (15), 190.1 (17), 189.1 (19), 177.0 (39), 164.1 (19), 152.0 (28), 151.0 (100), 123.0 (42), 109.0 (24), 107.0 (16).

^{1}H NMR (200 MHz, CDCl$_3$): δ 1.03 (s, 9H, H4'''), 2.19 (s, 2H, H2'''), 2.43 – 2.62 (m, 2H, H3, C4-CH), 2.62 – 2.82 (m, 1H, H4), 2.86 (dd, ^{2}J = 12.5 Hz, ^{3}J = 4.2 Hz, 1H, C4-CH), 3.77 (dd, ^{2}J = 8.6 Hz, ^{3}J = 6.3 Hz, 1H, H5), 3.86 (s, 3H, Ar''-OCH$_3$), 3.86 (s, 3H, Ar''-OCH$_3$), 4.06 (dd, ^{2}J = 8.6 Hz, ^{3}J = 6.2 Hz, 1H, H5), 4.16 (dd, ^{2}J = 11.1 Hz, ^{3}J = 7.2 Hz, 1H, C3-CH), 4.37 (dd, ^{2}J = 11.3 Hz, ^{3}J = 6.9 Hz, 1H, C3-CH), 4.85 (d, ^{3}J = 6.1 Hz, 1H, H2), 6.64 – 6.84 (m, 3H, Ar''-H), 6.96 – 7.09 (m, 2H, H3', H5'), 7.23 – 7.34 (m, 2H, H2', H6').

^{13}C NMR (50 MHz, CDCl$_3$): δ 29.8 (q, 3 x C4'''), 30.9 (s, C3'''), 33.1 (t, C4-C), 42.6 (d, C4), 48.1 (t, C2'''), 49.5 (d, C3), 56.00 (q, Ar''-OCH$_3$), 56.04 (q, Ar''-OCH$_3$), 62.2 (t, C3-C), 72.9 (t, C5), 82.7 (d, C2), 111.5 (d, C5''*), 112.0 (d, C2''*), 115.4 (dd, C3', C5', $^{2}J_{C\text{-}F}$ = 21.4 Hz), 120.6 (d, C6''), 127.5 (dd, C2', C6', $^{3}J_{C\text{-}F}$ = 8.1 Hz), 132.6 (s, C1''), 138.5 (d, C1', $^{4}J_{C\text{-}F}$ = 3.1 Hz), 147.7 (s, C4''), 149.1 (s, C3''), 162.4 (d, C4', $^{1}J_{C\text{-}F}$ = 245.5 Hz), 172.4 (s, C1''').

((2*S*,3*R*,4*R*)-4-(3,4-Dimethoxybenzyl)-2-(4-fluorophenyl)tetrahydrofuran-3-yl)methyl-2-ethylbutanoate (**15)**

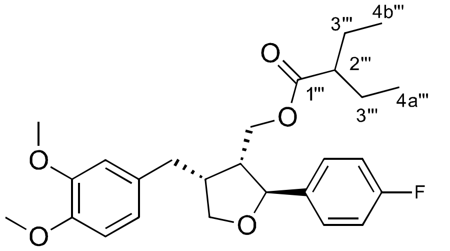

Analogous to **1**, using starting material **19** (15.2 mg, 0.044 mmol, 1.00 equiv.), 2-ethylbutanoic acid (11.7 mg, 0.101 mmol, 2.3 equiv.), EDCI.HCl (16.8 mg, 0.088 mmol, 2.0 equiv.), 4-DMAP (0.5 mg, 4.4 µmol, 0.1 equiv.) and DIPEA (19 µL, 0.110 mmol, 2.5 equiv.), and stirring for 13 in place of 16 h. The reaction solution was used directly for flash column chromatography (9 g silica, flow rate 20 mL / min, EtOAc / LP, 0 : 100 to 10 : 90 in 60 min) to afford the title compound **15**.

**Yield**:	16.2 mg, 83 %
**Appearance**:	colorless oil
***R*$_f$ (silica)**:	0.66 (EtOAc / heptane, 1 : 1)
**$[\alpha]_D^{23}$**:	+14.2 (c 0.65, MeOH)
**LC-HRMS (APCI)**:	calculated for M+Na$^+$: 467.2204, found: 467.2197, Δ: -1.50 ppm
**(log *P*)$_{calc}$**:	5.94 ± 0.49

GC-MS (EI, 70 eV, Method D): 11.51 min; 444.0 (M$^+$, 5), 194.0 (18), 190.1 (18), 189.1 (18), 178.1 (15), 177.0 (42), 164.1 (19), 163.1 (15), 152.1 (28), 151.0 (100), 123.0 (46), 109.0 (23), 107.0 (16).

^{1}H NMR (200 MHz, CDCl$_3$): δ 0.89 (t, ^{3}J = 7.4 Hz, 3H, H4a'''*), 0.90 (t, ^{3}J = 7.4 Hz, 3H, H4b'''*), 1.44 – 1.70 (m, 4H, H3'''), 2.14 – 2.29 (m, 1H, H2'''), 2.45 – 2.61 (m, 2H, H3, C4-CH), 2.62 – 2.81 (m, 1H, H4), 2.87 (dd, ^{2}J = 12.5 Hz, ^{3}J = 4.1 Hz, 1H, C4-CH), 3.77 (dd, ^{2}J = 8.6 Hz, ^{3}J = 6.3 Hz, 1H, H5), 3.86 (s, 6H, Ar''-OCH$_3$), 4.06 (dd, ^{2}J = 8.6 Hz, ^{3}J = 6.2 Hz, 1H, H5), 4.17 (dd, ^{2}J = 11.2 Hz, ^{3}J = 7.2 Hz, 1H, C3-CH), 4.41 (dd, ^{2}J = 11.3 Hz, ^{3}J = 6.7 Hz, 1H, C3-CH), 4.85 (d, ^{3}J = 6.1 Hz, 1H, H2), 6.65 – 6.84 (m, 3H, Ar''-H), 6.97 – 7.09 (m, 2H, H3', H5'), 7.24 – 7.34 (m, 2H, H2', H6').

^{13}C NMR (50 MHz, CDCl$_3$): δ 12.0 (q, C4a'''*), 12.1 (q, C4b'''*), 25.1 (t, 2 x C3'''), 33.2 (t, C4-C), 42.8 (d, C4), 49.1 (d, C2'''), 49.6 (d, C3), 56.0 (q, Ar''-OCH$_3$), 56.1 (q, Ar''-OCH$_3$), 62.3 (t, C3-C), 72.9 (t, C5), 82.7 (d, C2), 111.5 (d, C5''*), 112.0 (d, C2''*), 115.4 (dd, C3', C5', $^2J_{C-F}$ = 21.4 Hz), 120.6 (d, C6''), 127.5 (dd, C2', C6', $^3J_{C-F}$ = 8.1 Hz), 132.6 (s, C1''), 138.5 (d, C1', $^4J_{C-F}$ = 3.0 Hz), 147.7 (s, C4''), 149.1 (s, C3''), 162.4 (d, C4', $^1J_{C-F}$ = 245.5 Hz), 176.2 (s, C1''').

((2*S*,3*R*,4*R*)-4-(3,4-Dimethoxybenzyl)-2-(4-fluorophenyl)tetrahydrofuran-3-yl)methyl cyclopentanecarboxylate (**16**)

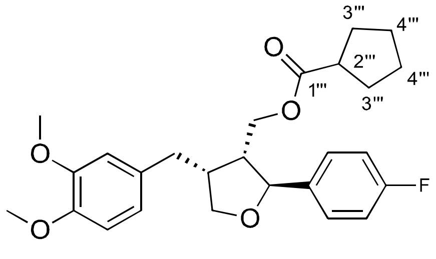

Analogous to **1**, using starting material **19** (31.2 mg, 0.090 mmol, 1.00 equiv.), cyclopentanecarboxylic acid (23.6 mg, 0.207 mmol, 2.3 equiv.), EDCI.HCl (34.5 mg, 0.180 mmol, 2.0 equiv.), 4-DMAP (1.1 mg, 9.0 µmol, 0.1 equiv.) and DIPEA (39 µL, 0.23 mmol, 2.5 equiv.). The reaction solution was used directly for flash column chromatography (9 g silica, flow rate 20 mL / min, EtOAc / LP, 0 : 100 to 15 : 85 in 16 min, then to 22 : 78 in 4 min) to afford the title compound **16**.

**Yield**:	38.2 mg, 96 %
**Appearance**:	colorless oil
***R*$_f$ (silica)**:	0.73 (EtOAc / LP, 1 : 1)
**$[\alpha]_D^{20}$**:	+13.8 (c 2.46, MeOH)
**LC-HRMS (ESI)**:	calculated for M+Na$^+$: 465.2048, found: 465.2024, Δ: -5.16 ppm
**(log *P*)$_{calc}$**:	5.50 ± 0.49

GC-MS (EI, 70 eV, Method C): 16.70 min; 442.3 (M$^+$, 4), 194.1 (22), 190.1 (22), 189.1 (21), 177.1 (41), 164.1 (23), 163.1 (15), 152.1 (30), 151.1 (100), 123.0 (48), 109.1 (32), 107.1 (18).

^{1}H NMR (200 MHz, CDCl$_3$): δ 1.47 – 1.98 (m, 8H, 4 x H3''', 4 x H4'''), 2.44 – 2.79 (m, 4H, H3, H4, C4-CH, H2'''), 2.85 (dd, ^2J = 12.4 Hz, ^3J = 4.3 Hz, 1H, C4-CH), 3.76 (dd, ^2J = 8.5 Hz, ^3J = 6.4 Hz, 1H, H5), 3.86 (s, 3H, Ar''-OCH$_3$), 3.87 (s, 3H, Ar''-OCH$_3$), 4.07 (dd, ^2J = 8.6 Hz, ^3J = 6.2 Hz, 1H, H5), 4.18 (dd, ^2J = 11.2 Hz, ^3J = 7.4 Hz, 1H, C3-CH), 4.38 (dd, ^2J = 11.2 Hz, ^3J = 6.6 Hz, 1H, C3-CH), 4.85 (d, ^3J = 6.0 Hz, 1H, H2), 6.65 – 6.75 (m, 2H, H2'', H6''*), 6.80 (d, ^3J = 7.9 Hz, 1H, H5''*), 7.02 (dd, ^3J = 8.7 Hz, $^3J_{H-F}$ = 8.7 Hz, 2H, H3', H5'), 7.29 (dd, ^3J = 8.6 Hz, $^4J_{H-F}$ = 5.4 Hz, 2H, H2', H6').

^{13}C NMR (50 MHz, CDCl$_3$): δ 25.9 (t, 2 x C4'''), 30.1 (t, 2 x C3'''), 33.2 (t, C4-C), 42.7 (d, C4), 43.9 (d, C2'''), 49.5 (d, C3), 55.98 (q, Ar''-OCH$_3$), 56.00 (q, Ar''-OCH$_3$), 62.5 (t, C3-C), 72.9 (t, C5), 82.7 (d, C2), 111.4 (d, C5''*), 111.9 (d, C2''*), 115.4 (dd, C3', C5', $^2J_{C-F}$ = 21.5 Hz), 120.5 (d, C6''), 127.5 (dd, C2', C6', $^3J_{C-F}$ = 8.1 Hz), 132.6 (s, C1''), 138.5 (d, C1', $^4J_{C-F}$ = 3.1 Hz), 147.7 (s, C4''), 149.1 (s, C3''), 162.3 (d, C4', $^1J_{C-F}$ = 245.4 Hz), 176.7 (s, C1''').

((2*S*,3*R*,4*R*)-4-(3,4-Dimethoxybenzyl)-2-(4-fluorophenyl)tetrahydrofuran-3-yl)methyl cyclohexanecarboxylate (**17**)

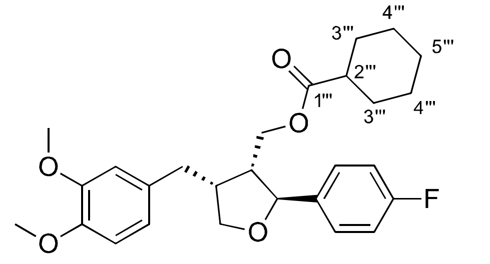

Analogous to **1**, using starting material **19** (31.2 mg, 0.090 mmol, 1.00 equiv.), cyclohexanecarboxylic acid (26.5 mg, 0.207 mmol, 2.3 equiv.), EDCI.HCl (34.5 mg, 0.180 mmol, 2.0 equiv.), 4-DMAP (1.1 mg, 9.0 µmol, 0.1 equiv.) and DIPEA (39 µL, 0.23 mmol, 2.5 equiv.). The reaction solution was used directly for flash column chromatography (9 g silica, flow rate 20 mL / min, EtOAc / LP, 0 : 100 to 15 : 85 in 16 min, then to 22 : 78 in 4 min) to afford the title compound **17**.

Yield: 37.2 mg, 91 %

Appearance: colorless oil

***R*f (silica)**: 0.76 (EtOAc / LP, 1 : 1)

$[\alpha]_D^{20}$: +15.1 (c 2.10, MeOH)

LC-HRMS (ESI): calculated for $M+Na^+$: 479.2204, found: 479.2185, Δ: -3.96 ppm

(log *P*)calc: 6.06 ± 0.49

GC-MS (EI, 70 eV, Method D): 19.53 min; 456.3 (M^+, 4), 194.1 (24), 190.1 (24), 189.1 (23), 177.1 (44), 164.1 (26), 163.1 (16), 152.1 (30), 151.1 (100), 123.0 (47), 109.1 (33), 107.1 (18).

^{1}H NMR (200 MHz, $CDCl_3$): δ 1.11 – 1.95 (m, 10H, 4 x H3''', 4 x H4''', 2 x H5'''), 2.17 – 2.34 (m, 1H, H2'''), 2.42 – 2.62 (m, 2H, H3, C4-CH), 2.62 – 2.79 (m, 1H, H4), 2.85 (dd, ^2J = 12.4 Hz, ^3J = 4.3 Hz, 1H, C4-CH), 3.77 (dd, ^2J = 8.6 Hz, ^3J = 6.3 Hz, 1H, H5), 3.86 (s, 3H, Ar''-OCH_3), 3.87 (s, 3H, Ar''-OCH_3), 4.07 (dd, ^2J = 8.6 Hz, ^3J = 6.3 Hz, 1H, H5), 4.17 (dd, ^2J = 11.3 Hz, ^3J = 7.3 Hz, 1H, C3-CH), 4.38 (dd, ^2J = 11.3 Hz, ^3J = 6.7 Hz, 1H, C3-CH), 4.84 (d, ^3J = 6.2 Hz, 1H, H2), 6.65 – 6.75 (m, 2H, H2'', H6''*), 6.81 (d, ^3J = 7.9 Hz, 1H, H5''*), 7.02 (dd, ^3J = 8.7 Hz, $^3J_{H-F}$ = 8.7 Hz, 2H, H3', H5'), 7.29 (dd, ^3J = 8.6 Hz, $^4J_{H-F}$ = 5.4 Hz, 2H, H2', H6').

^{13}C NMR (50 MHz, $CDCl_3$): δ 25.5 (t, 2 x C4'''), 25.8 (t, C5'''), 29.1 (t, 2 x C3'''), 29.1 (t, C3'''), 33.2 (t, C4-C), 42.7 (d, C4), 43.3 (d, C2'''), 49.5 (d, C3), 55.97 (q, Ar''-OCH_3), 56.00 (q, Ar''-OCH_3), 62.3 (t, C3-C), 72.9 (t, C5), 82.7 (d, C2), 111.4 (d, C5''*), 111.9 (d, C2''*), 115.4 (dd, C3', C5', $^2J_{C-F}$ = 21.4 Hz), 120.5 (d, C6''), 127.5 (dd, C2', C6', $^3J_{C-F}$ = 8.1 Hz), 132.6 (s, C1''), 138.5 (d, C1', $^4J_{C-F}$ = 3.1 Hz), 147.7 (s, C4''), 149.1 (s, C3''), 162.3 (d, C4', $^1J_{C-F}$ = 245.7 Hz), 176.0 (s, C1''').

References

[1] K. Schwetlick, Organikum, Wiley, 2009.

[2] Y. Gao, J.M. Klunder, R.M. Hanson, H. Masamune, S.Y. Ko, K.B. Sharpless, Catalytic asymmetric epoxidation and kinetic resolution: modified procedures including in situ derivatization, Journal of the American Chemical Society, 109 (1987) 5765-5780.
[3] S.P. Hehner, T.G. Hofmann, W. Dröge, M.L. Schmitz, The antiinflammatory sesquiterpene lactone parthenolide inhibits NF-kappa B by targeting the I kappa B kinase complex, J Immunol, 163 (1999) 5617-5623.
[4] N. Fakhrudin, B. Waltenberger, M. Cabaravdic, A.G. Atanasov, C. Malainer, D. Schachner, E.H. Heiss, R. Liu, S.M. Noha, A.M. Grzywacz, J. Mihaly-Bison, E.M. Awad, D. Schuster, J.M. Breuss, J.M. Rollinger, V. Bochkov, H. Stuppner, V.M. Dirsch, Identification of plumericin as a potent new inhibitor of the NF-kappaB pathway with anti-inflammatory activity in vitro and in vivo, Br J Pharmacol, 171 (2014) 1676-1686.
